# *GhCIPK6a* increases salt tolerance in transgenic upland cotton by involving in ROS scavenging and MAPK signaling pathways

**DOI:** 10.1186/s12870-020-02548-4

**Published:** 2020-09-14

**Authors:** Ying Su, Anhui Guo, Yi Huang, Yumei Wang, Jinping Hua

**Affiliations:** 1 Laboratory of Cotton Genetics; Genomics and Breeding / Key Laboratory of Crop Heterosis and Utilization of Ministry of Education, Ministry of Education /Beijing Key Laboratory of Crop Genetic Improvement, College of Agronomy and Biotechnology, China Agricultural University, No. 2, Yuanmingyuan West Rd, Haidian District, Beijing, 100193 China; 2 Oil Crops Research Institute, Chinese Academy of Agricultural Sciences, Wuhan, 430062 Hubei China; 3 Research Institute of Cash Crops, Hubei Academy of Agricultural Sciences, Wuhan, 430064 Hubei China

**Keywords:** CIPK, Salt stress, Co-expression, Upland cotton, Signaling pathway

## Abstract

**Background:**

Salt stress is one of the most damaging abiotic stresses in production of Upland cotton (*Gossypium hirsutum*). Upland cotton is defined as a medium salt-tolerant crop. Salinity hinders root development, shoots growth, and reduces the fiber quality.

**Results:**

Our previous study verified a *GhCIPK6a* gene response to salt stress in *G. hirsutum*. The homologs of *GhCIPK6a* were analyzed in A_2_ (*G. arboreum*), D_5_ (*G*. *raimondii*), and AD_1_ (*G. hirsutum*) genomes. GhCIPK6a localized to the vacuole and cell membrane. The GhCBL1-GhCIPK6a and GhCBL8-GhCIPK6a complexes localized to the nucleus and cytomembrane. Overexpression of *GhCIPK6a* enhanced expression levels of co-expressed genes induced by salt stress, which scavenged ROS and involved in MAPK signaling pathways verified by RNA-seq analysis. Water absorption capacity and cell membrane stability of seeds from *GhCIPK6a* overexpressed lines was higher than that of wild-type seeds during imbibed germination stage. The seed germination rates and seedling field emergence percentages of *GhCIPK6a* overexpressed lines were higher than that of control line under salt stress. Moreover, overexpressing of *GhCIPK6a* in cotton increased lint percentage, and fiber length uniformity under salt stress.

**Conclusions:**

We verified the function of *GhCIPK6a* by transformation and RNA-seq analysis. *GhCIPK6a* overexpressed lines exhibited higher tolerance to abiotic stresses, which functioned by involving in ROS scavenging and MAPK pathways. Therefore, *GhCIPK6a* has the potential for cotton breeding to improve stress-tolerance.

## Background

Soil salinity is one of the serious abiotic stresses that affect crop plant growth and development, and eventually reduce the yield and descend the quality [1, 2]. Plants initiate a series of adaptive mechanism and survival responses to abiotic stresses, and result in serial changes in gene expression [3–6]. These stress-response genes involve Ca^2+^-sensors [7, 8], transcription factors [9, 10], protein kinases [11–13], osmotic proteins [14–16], and hydroperoxidases [17, 18], in which protein kinases play a particularly important role in plant response. A group of serine-threonine kinases, designated as CBL-interacting protein kinases (CIPKs), which interact specifically with calcineurin B-like proteins (CBLs), has been characterized in plant genomes [19, 20].

CIPK proteins consist of a conserved N-terminal kinase catalytic domain, and a C-terminal regulatory domain separated from the kinase domain by a variable junction domain and the N-terminal kinase domain contains a putative activation loop phosphorylated by other protein kinases [21]. The highly conserved NAF/FISL domain in C-terminal of CIPK proteins is required and sufficient for interacting with CBL proteins, and functions as an auto-inhibitory domain [22]. Another motif, protein phosphatase interaction (PPI), is necessary for the interaction with abscisic acid-insensitive (ABI) protein [23]. The PPI motif controls the phosphorylation status of CIPK and PP2Cs (protein phosphatase 2C) [23–25]. CBLs generally interact with CIPKs to form CBL-CIPK complexes that regulate downstream target proteins. CIPK proteins and CBL-CIPK complexes were involved in various responsive processes in Arabidopsis and other plants, such as rice (*Oryza sativa*) [26–29], maize (*Zea mays*) [30–32], *Populus euphratica* [33, 34], canola (*Brassica napus*) [35, 36], eggplant (*Solanum melongena*) [37], tomato (*Solanum lycopersicum*) [38], foxtail millet (*Setaria italica* (L.) P. Beauv) [39], pineapple (*Ananas comosus*) [40] and cotton [41–44]. CIPKs and CBL-CIPK complex have been implicated in the plant’s response to abiotic stresses, biotic stresses, phytohormones, and nutrient deprivation [45–50].

In Arabidopsis, overexpression of *AtCIPK6* gene increased the tolerance to salt stress [51, 52]. AtCBL4/SOS3 interacts with AtCIPK24/SOS2 in the roots to mediate Na^+^ extrusion via the action of the H^+^/Na^+^ antiporter SOS1 at the plasma membrane in SOS signaling pathway in *Arabidopsis* under salt stress [53, 54]. There were a novel SOS pathway, CBL10–CIPK8–SOS1, functions to transport accumulated Na^+^ out of cells to regulate salt tolerance [50]. The AtCBL1/9-AtCIPK23 complex activates the AKT1 channel at the plasma membrane to enhance K^+^ uptake under low K^+^ condition [24, 55, 56]. Meanwhile, AtCIPK23 and AtKC1 act synergistically to modulate the activity of AKT1 [57]. AtCBL9-AtCIPK23 complex was shown to interact with the nitrate transporter NRT1.1 during the primary nitrate response [49, 58, 59].

Upland cotton (*G. hirsutum*) is defined as a medium salt tolerant crop [60, 61]. Salinity hinders root development, shoots growth, and reduces the fiber quality [62]. Newly released *Gossypium* species genomes provide novel platform for functional genomics research [63–69]. *GhCIPK* homolog, *GhCIPK1* is involved in the cotton fiber elongation process [70], and is shown to be co-expressed with and preferentially interact with GhCBL2/3 [71]. Heterologous expression of *GhCIPK6* (KC465063) in *Arabidopsis* enhanced tolerance to salt treatment, and in transgenic cotton, this gene mediates the uptake of K^+^ under Ca^2+^ − deficient condition [42, 44].

In our previous study, we characterized a salt-responsive gene from cDNA libraries and Microarray results, *GhCIPK6a* (Accession number in GenBank: HM002633), homologous to *AtCIPK6* [41]. *GhCIPK6a* also differently expressed in salt-tolerant and salt-sensitive Upland cotton varieties under salt stress by transcriptome analysis [72]. We isolated the full length of the *GhCIPK6a* gene and characterized its function in Upland cotton. Changes in physiological indexes showed that over-expression of *GhCIPK6a* in Upland cotton significantly strengthened the salt tolerance, which were verified that overexpressed *GhCIPK6a* enhanced co-expressed genes expression levels in stress signaling pathways by RNA-seq results. Meanwhile, the transgenic cotton grew better than wild type in saline field. Here we elucidate the function of *GhCIPK6a* and the potential use in transgenic cotton breeding.

## Results

### Isolation and basic analysis of *GhCIPK6a*

In present study, cDNA and genomic DNA sequences of *GhCIPK6a* were isolated from Upland cotton ‘Zhong G5’ [41]. A comparison of the genomic DNA and cDNA sequences using GSDS (http://gsds.cbi.pku.edu.cn/) [73] revealed that there was no intron in the genomic sequence of *GhCIPK6a*.

**Fig. 1:**
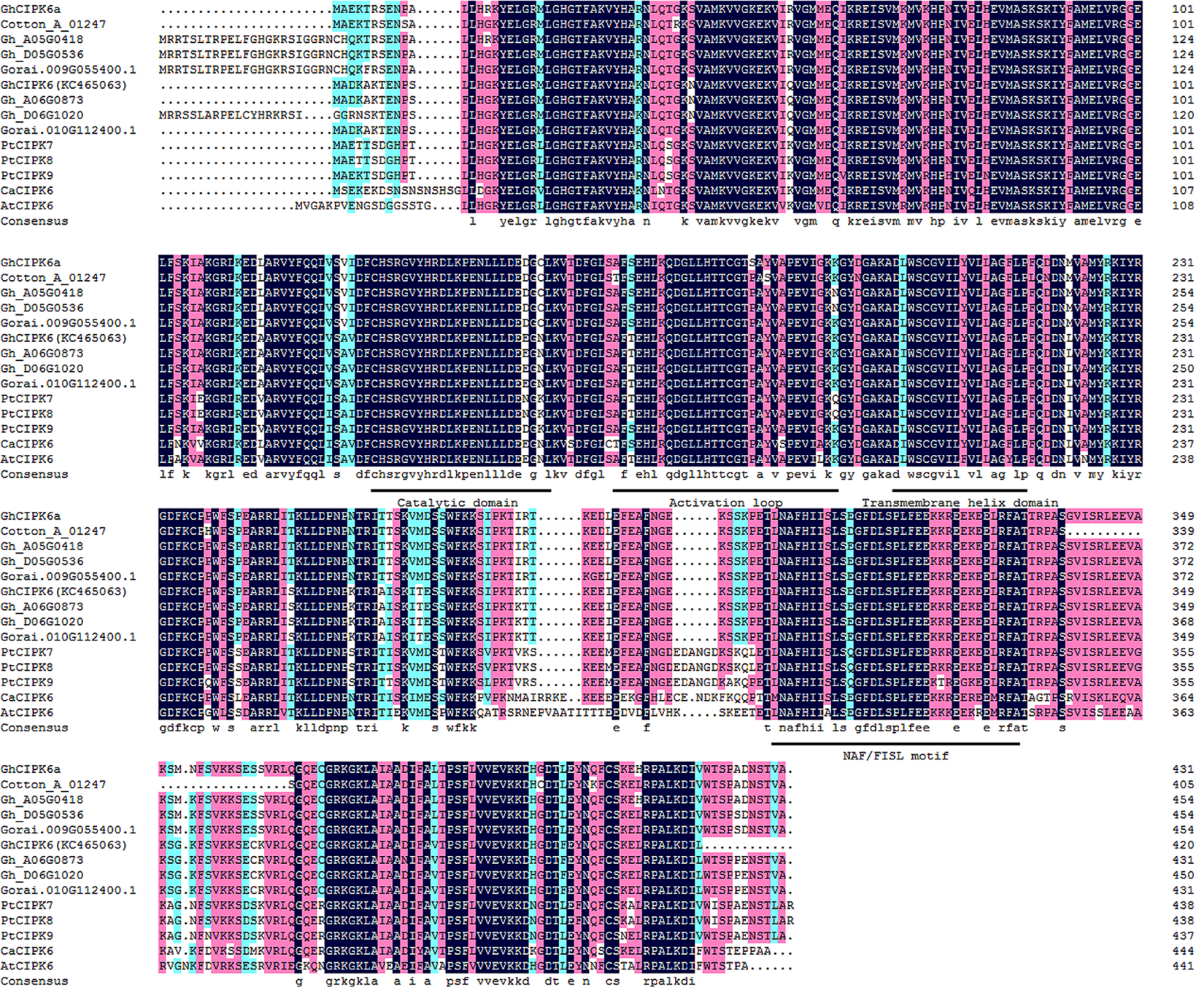
Multiple sequence alignment of CIPK proteins using ClustalW implemented in DNAMAN. Conserved functional domains are underlined. At, *Arabidopsis thaliana*; Ca, *Cicer arietinum*; Pt, *Populus trichocarpa*; Gh, *G. hirsutum*; Cotton_A, *G. arboreum*; Gorai, *G. raimondii*. Numbers shown in the right indicate amino acid residue positions

*GhCIPK6a* contained an open reading frame (ORF) of 1296 bp and encoded a protein of 431 amino acids and molecular weight 48.637 kD. Using ScanProsite online software (http://au.expasy.org/tools/scanprosite/), motif scan analysis showed that GhCIPK6a included a Serine/Threonine protein kinase catalytic domain at the N-terminal, which contained an ATP-binding region and an active site, and a CBL-interacting NAF/FISL module at the C-terminal. GhCIPK6a contains a transmembrane helix domain between amino acid residues 198 and 217 (Fig. 1).

To determine genomic location of *GhCIPK6a*, we compared *GhCIPK6a* to the genome sequences of D_5_ (*G. raimondii*) [65], A_2_ (*G. arboreum*) [63], and AD_1_ (*G. hirsutum* L*.* acc. TM-1) [66], and searched for homologs (Table 1). We identified four homologs in the D_5_ genome, including *Gorai.001G032000.1*, *Gorai.009G055400.1*, *Gorai.010G112400.1*, and *Gorai.013G216700.1*, in which, *Gorai.009G055400.1* was the most identified to *GhCIPK6a* (Additional file 6 Fig. S1). In the A_2_ genome sequence, three homologs of *GhCIPK6a* were identified, including *Cotton_A_01247*, *Cotton_A_09063*, and *Cotton_A_07248*, and *GhCIPK6a* exhibited the highest similarity to *Cotton_A_01247*. The AD_1_ genome sequence harbored three homologs of *GhCIPK6a*, which genes ID was *Gh_A05G0418*, *Gh_D05G0536*, and *Gh_D13G1983*. Gh_A05G0418 and Gh_D05G0536 shared more than 99% amino acid sequence identity with GhCIPK6a. Phylogenetic analysis showed that all the CIPKs were classified as four groups (Group I to IV, Additional file 6 Fig. S1). GhCIPK6a was classified into Group III. The nucleotide sequence of *GhCIPK6a* shared 66.22% identity with *AtCIPK6* (*AT4G30960*, http://www.arabidopsis.org/), and 70.72% with *CaCIPK6* (EU492906.1, *Cicer arietinum*, http://www.ncbi.nlm.nih.gov/). Meanwhile, multiple sequence alignment of GhCIPK6 with related proteins were carried out (Fig. 1). These CIPK protein kinases included highly conserved functional domains, such as catalytic domain, activation loop, transmembrane helix domain, and NAF/FISL motif.

**Table 1:** Characteristics of homologous genes in the A_2_, D_5_, and AD_1_ genomes

Gene ID/Gene name	Genomic position	ORF length (bp)	DNA length (bp)	Protein length (aa)	Protein molecular mass (kd)	pI	Reference
GhCIPK6a *(*HM002633)	–	1296	1296	431	48.71	9.00	Zhang et al. 2011 [41]
Cotton_A_01247	Ca03:32871827:32873122^a^	1218	1296	405	45.80	9.04	Li et al. 2014 [63]
Gorai.009G055400.1	Chr09:4028418:4029980^a^	1365	1563	454	51.29	9.35	Paterson et al. 2012 [65]
Gh_A05G0418	A05:4720527:4722095^a^	1365	1596	454	51.41	9.31	Zhang et al. 2015 [66]
Gh_D05G0536	D05:4357435:4358998^a^	1365	1564	454	51.30	9.26	Zhang et al. 2015 [66]
GhCIPK6 (KC465063)	–	1296	–	431	47.33	9.09	He et al. 2013 [42]
Gorai.010G112400.1	Chr10:21475391:21476686^a^	1296	1296	431	48.49	9.04	Paterson et al. 2012 [65]
Gh_A06G0873	A06:33360018:33361313^a^	1296	1296	431	48.55	9.10	Zhang et al. 2015 [66]
Gh_D06G1020	D06:21731900:21733431^a^	1353	1532	450	50.79	9.31	Zhang et al. 2015 [66]

^a^, antisense strand; −-, no data

### Subcellular localization of GhCIPK6a

**Fig. 2:**
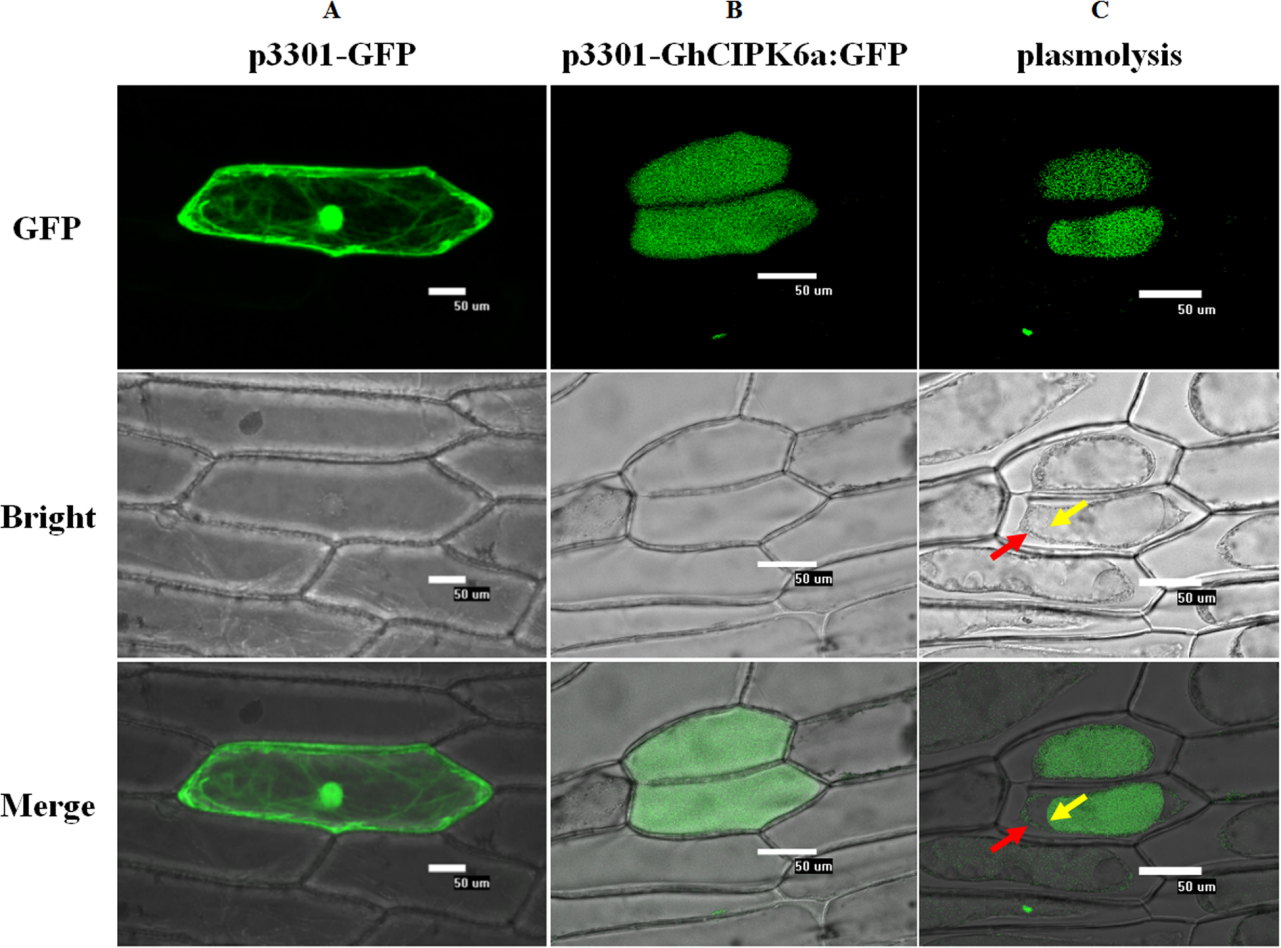
Subcellular localization of the GhCIPK6a:GFP fusion protein in onion epidermal cells. **a.** Fluorescence microscopy images of cells expressing GFP protein. **b.** Fluorescence microscopy images of cells expressing the GhCIPK6a:GFP fusion. **c.** Fluorescence microscopy images of plasmolyzed cells in 30% sucrose solution. The cell membrane is marked with a red arrow, and the vacuole membrane is marked with a yellow arrow. Confocal images of epidermal cells were captured 22–24 h after transformation. Bar = 50 μm

Subcellular localization analysis using SubLoc v1.0 (http://www.bioinfo.tsinghua.edu.cn/SubLoc/) indicated that GhCIPK6a was localized to the cytoplasm, which was confirmed by generating a construct by fusing GFP to C-terminal end of *GhCIPK6a* under the control of the CaMV 35S promoter, and transiently expressing the construct in onion epidermal cells. Indeed, fluorescence was specifically localized to the cytoplasm (Fig. 2b)*.* To establish whether the GhCIPK6a: GFP fusion was present at the cell membrane; the onion epidermal cells were plasmolyzed in sucrose solution. The analysis demonstrated that the fusion protein was restricted to the vacuole and cell membrane (Fig. 2c).

### Verification of the interaction between GhCIPK6a and GhCBLs in vivo

The activation of CIPKs was regulated by binding to one or more CBL proteins. It was previously reported that GhCIPK1 interacted with GhCBL2 and GhCBL3 [71]. So, we detected and investigated which GhCBL proteins interact with GhCIPK6a using the BiFC method*.*

**Fig. 3:**
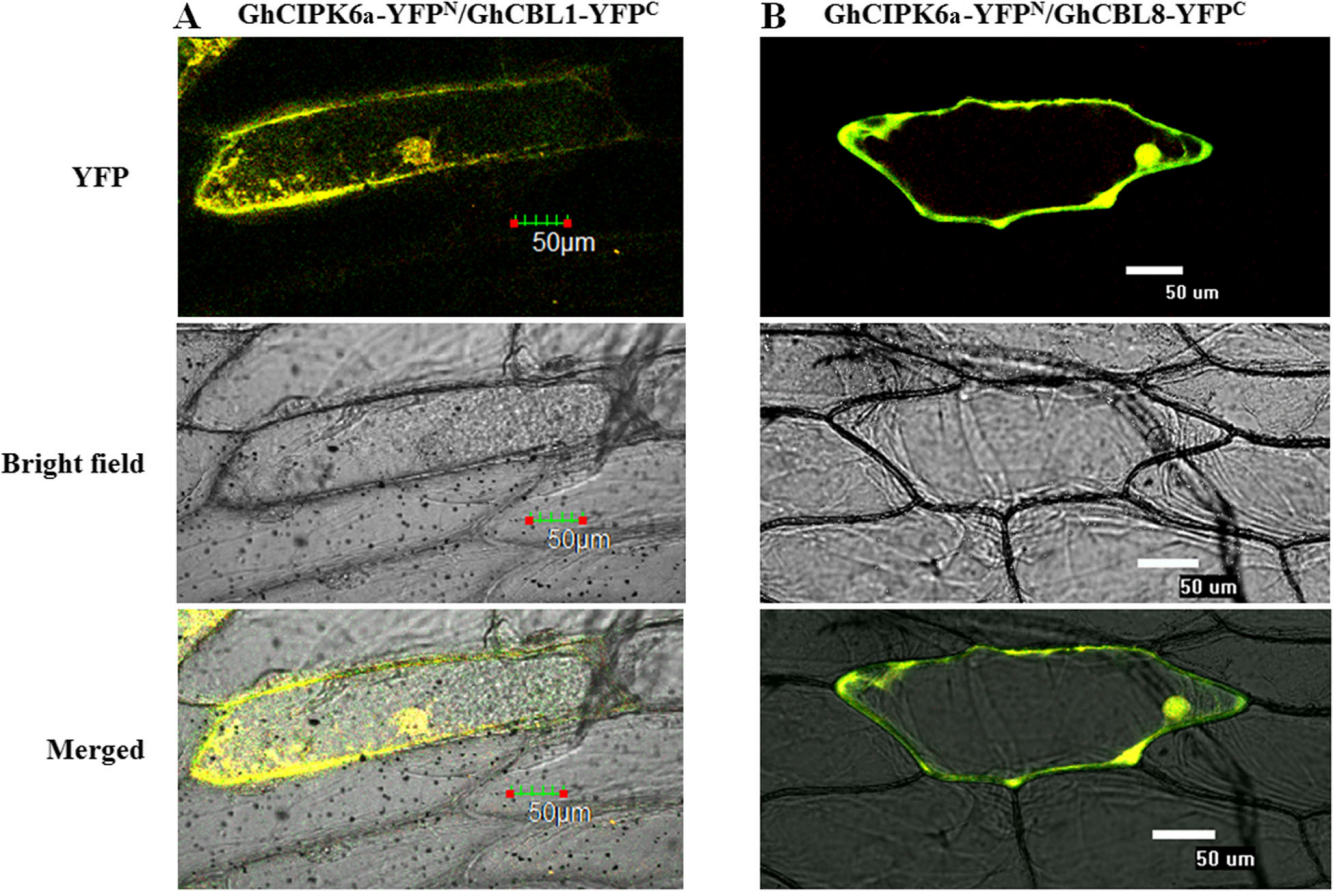
BiFC assay demonstrated the interaction between GhCIPK6a and GhCBLs in onion epidermal cells. GhCIPK6a interacted with GhCBL1 (**a**) and GhCBL8 (**b**) in vivo. Confocal images of epidermal cells were captured 22–24 h after transformation. Bar = 50 μm

Four GhCBL gene sequences obtained from NCBI (http://www.ncbi.nlm.nih.gov/): GhCBL1 (EU085038.1), GhCBL2 (EU085042.1), GhCBL3 (EU085040.1), and GhCBL8 (EU085041.1). When GhCIPK6a-YFP^N^ and GhCBLs-YFP^C^ fusion genes were co-expressed in onion epidermal cells using particle bombardment, yellow fluorescence signals were observed in the nucleus and cell membrane when GhCIPK6a co-expressed with GhCBL1 and GhCBL8 (Fig. 3). By contrast, no signal was observed when GhCIPK6a co-expressed with GhCBL2 or GhCBL3 in onion epidermal cells (data not shown).

### *GhCIPK6a* and salt-response genes significantly up regulated under salt treatment

In the salt-sensitive cultivar ‘Zhong G5’ [41], *GhCIPK6a* transcript accumulated to higher levels in the root, and expression level significantly increased by salt treatment for 1, 6, 12, and 24 h. In stem tissue, the *GhCIPK6a* expression was induced after a longer period of salt treatment than that in the root, and the fold-change was less than in the root (Additional file 7 Fig. S2a)*.*

**Fig. 4:**
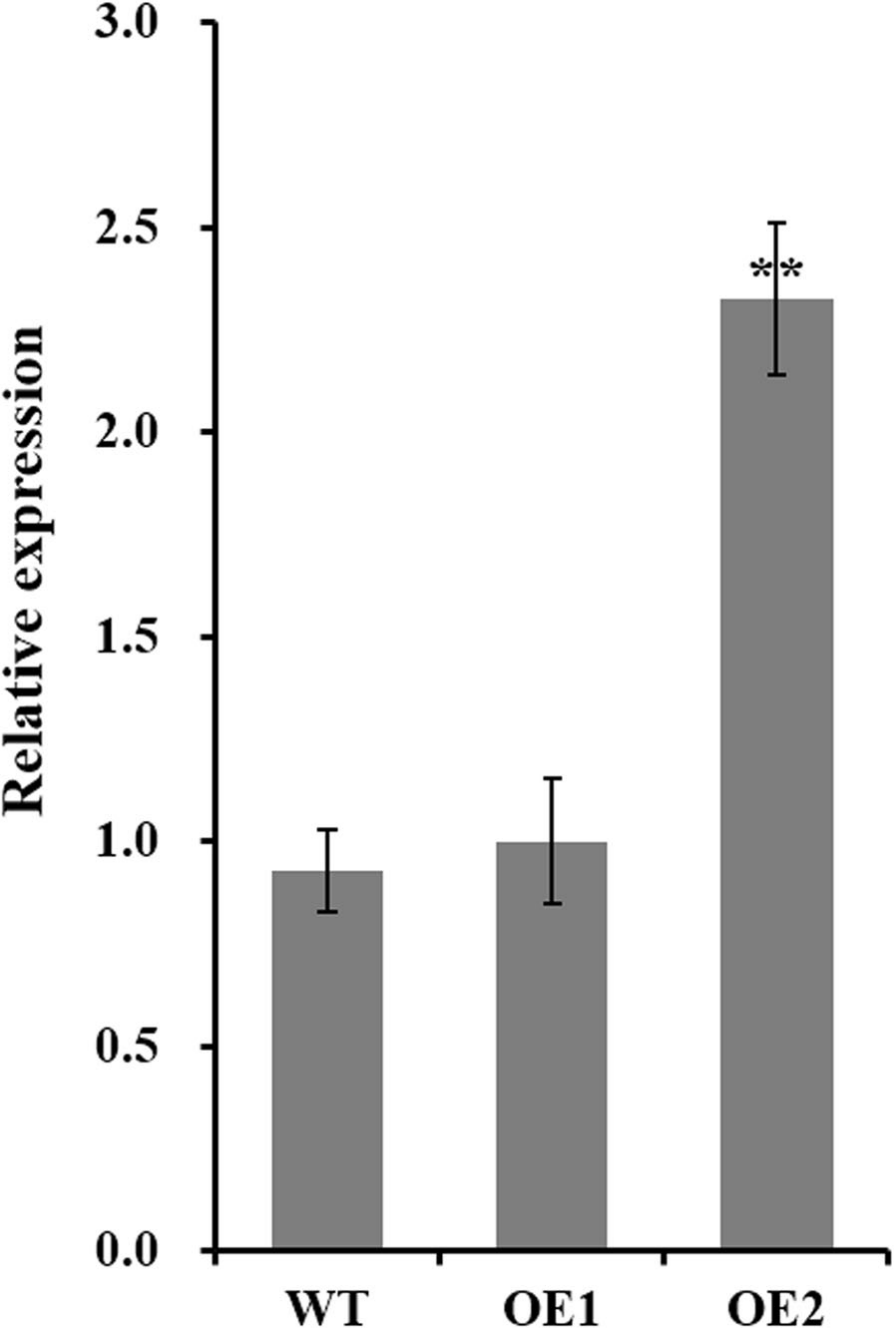
Relative expression of *GhCIPK6a* in the root tissues of transgenic lines (OE1 and OE2) and wild-type (WT) plants at the three-leaf stage under normal treatment. *, *p*-value < 0.05; **, *p*-value < 0.01

To investigate whether overexpression of *GhCIPK6a* in cotton enhanced salt tolerance, we transformed *GhCIPK6a* into Upland cotton cultivar ‘11–0516’. Eleven transgenic cotton individuals (T_1_ generation) were obtained (Additional file 8 Fig. S3a). Through kanamycin resistance assay, PCR analysis and Southern blotting assay, we obtained two T_2_ generation transgenic progeny plants that harbored two copies of insert fragment, which named by OE1 and OE2 (Additional file 8 Fig. S3b). We examined the expression level of GhCIPK6a in roots of transgenic and control plants at the three-leaf stage by qRT-PCR analysis. *GhCIPK6a* expression was significantly higher in OE2 than that in wild type line without treatment (Fig. 4). However, the expression level in OE1 line no significantly increased than wild-type cotton. Therefore, we further analyze the functions of *GhCIPK6a* gene using OE2 transgenic line*.*

**Fig. 5:**
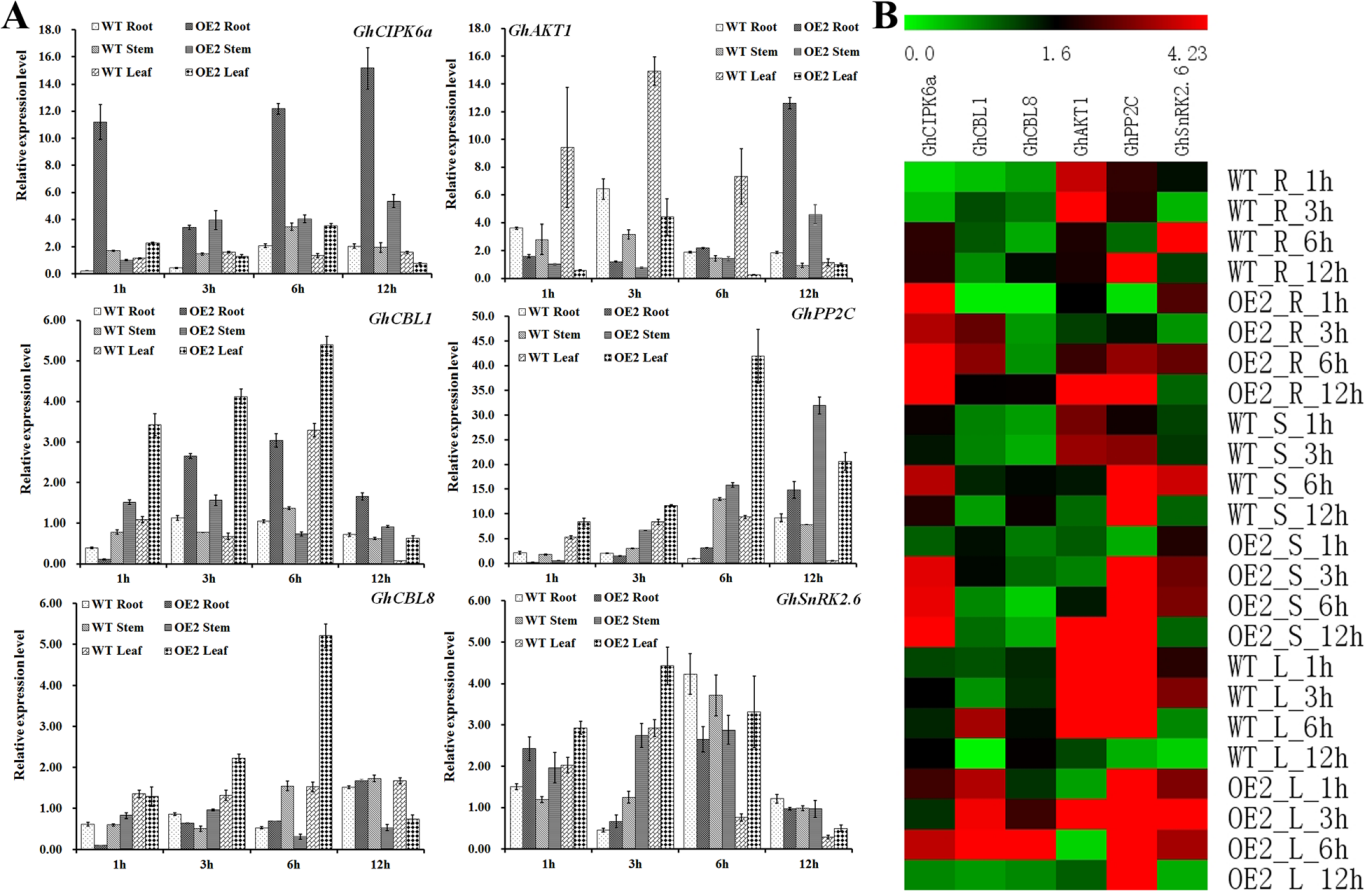
qRT-PCR analysis of *GhCIPK6a* expression and genes that were co-expressed with *GhCIPK6a* in different tissues under salt stress. **a.** Relative expression values of genes co-expressed with *GhCIPK6a*. **b.** Heatmap of *GhCIPK6a* expression and genes co-expressed with *GhCIPK6a* in different tissues under salt stress. WT, the wild type line, 11-0516; OE2, the transgenic cotton. R, root tissue; S, stem tissue; L, leaf tissue

We also examined the expression level of *GhCIPK6a* in different tissues of transgenic cotton (OE2) under salt stress (Additional file 7 Fig. S2b). Overexpressed *GhCIPK6a* strongly induced by salt stress in roots of transgenic cotton, especially after 1, 3, 6, and 12 h salt treatments. In addition, the expression profiles of *GhCIPK6a* gene in other tissues were similar to that of wild type. Moreover, *GhCIPK6a* expression increased with increasing duration of exposure to salt stress (Fig. 5). To determine the function of *GhCIPK6a* under multiple abiotic stresses, we analyzed the cotton expression profiles of *GhCIPK6a* using public datasets from PLEXdb and GEO. *GhCIPK6a* gene was analyzed under multiple abiotic stresses, such as ABA, cold, drought, salinity, and alkalinity (pH) in *G. hirsutum* (Additional file 9 Fig. S4).

CIPKs interact with CBLs and PP2Cs to form a complex that regulates the activity of K^+^ transporters [24, 74]. Therefore, we examined the expression level of *GhAKT1* in transgenic cotton and wild type under salt stress and control (in hydroponic growth, Fig. 5) conditions. *GhAKT1* expression only increased strongly in the root of the OE2 line at three-leaf stage after 12 h salt stress treatment, which exhibited similar tendency in stem. After salt treatment at three-leaf stage*, GhAKT1* expression was strongly induced in all tissues of the wild-type line*,* especially the leaf. The increase in expression level might be associated with maintaining K^+^ homeostasis in root upon exposure to salt stress, which would enhance the salt tolerance of transgenic line in turn.

GhCIPK6a interacts with GhCBL1/GhCBL8, SnRK2.6 and PP2C proteins, respectively, to regulate the expression of downstream genes [24, 74]. Then, we analyzed the expression profiles of *GhCBL1*, *GhCBL8*, *GhPP2C* (DQ303437.1), and *GhSnRK2.6* (JN872373) [12, 75] (Fig. 5). The transcript levels of *GhCBL1* and *GhCBL8* rose sharply soon after exposure to salt stress, and then decreased at 12 h after salt stress in leaves. During the same time, *GhCBL1* expression was induced by salt stress in the stem of the transgenic line, OE2. *GhPP2C* was upregulated in all tissues of OE2 after salt treatment, especially the leaves, except in the roots. *GhSnRK2.6* was strongly induced in all tissues of OE2 at one and three hours after treatment, but transcripts only accumulated in the leaves after 6 and 12 h stresses (Fig. 5)*.*

### *GhCIPK6a* enhanced salt tolerance during germination and seedling stages

**Fig. 6:**
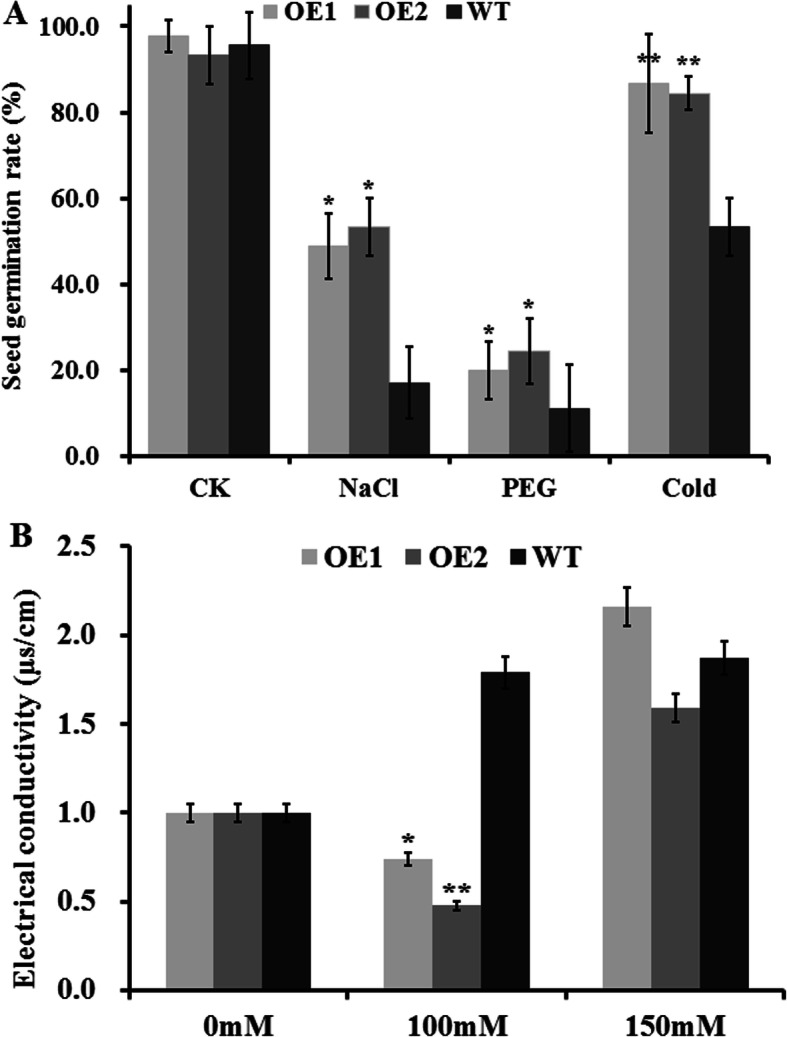
Analysis of seed germination rate and electrical conductivity of transgenic lines (OE1 and OE2) and wild-type (WT) line under abiotic stresses. **a**. Seed germination rate of transgenic lines (OE1 and OE2) and wild-type (WT) line under salt, PEG, and cold treatments. **b**. Electrical conductivity analysis of transgenic lines (OE1 and OE2) and wild-type (WT) line after soaking for 24 h in 0, 100, and 150 mmol·L^− 1^ NaCl solution. *, *p*-value < 0.05; **, *p*-value < 0.01

Under salt stress, germination and emergence of cotton were the key stages. We determined the germination potential and seed germination rate of cotton seed using rolls of filter paper placed upright under salt treatment (150 mmol·L^− 1^ NaCl) and control (distilled water, CK) (Fig. 6a, Additional file 10 Fig. S5)*.* The seed germination rate of the transgenic cotton lines (48.89 and 53.33%) was significantly higher than that of wild type (17.12%) under salt stress (Fig. 6a, Additional file 10 Fig. S5). But the differences between transgenic lines and wild type line in germination potential under salt stress significantly (data not shown)*.* In addition, we also obtained the homozygotes of transgenic Arabidopsis, analyzed seed germination rate and growth under salt treatment. The transgenic Arabidopsis maintained higher seed germination rate and grew normal under NaCl concentration up to 200 mmol·L^− 1^ as well (data not shown).

**Fig. 7:**
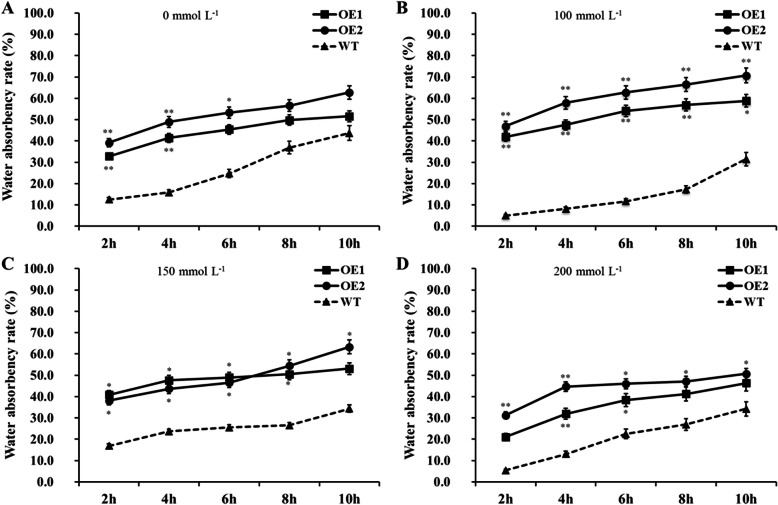
The water absorbency rate in imbibed germination stage of transgenic lines (OE1 and OE2) and wild-type (WT) line exposed to solution containing different NaCl concentrations (mmol·L^− 1^). **a**, control, represented the water absorbency rates of transgenic lines and wild type line exposed to distilled water (0 mmol·L^− 1^ NaCl concentration). B, C, and D represented the water absorbency rates of transgenic lines and wild type line exposed to solution with different NaCl concentrations, (**b**) 100 mmol·L^− 1^, (**c**) 150 mmol·L^− 1^, and (**d**) 200 mmol·L^− 1^, respectively. *, *p*-value < 0.05; **, *p*-value < 0.01

Meanwhile, we analyzed the changes in water absorbency rate of transgenic and wild type cotton seeds in imbibed germination stage (Fig. 7), and the effect of salt stress on cell membrane permeability by determining the electrical conductivity after soaking for 24 h in different concentrations of NaCl solution (Fig. 6b). The cell membrane of the transgenic lines (OE1 and OE2) was more stable than that of wild type, and water absorption capacity was higher than that of wild type during imbibed germination stage. Thus, transgenic cottonseeds maintain a higher seed germination rate under salt stress due to increased stability of cell membrane, which ensured that water was absorbed at normal rates under salt stress. Therefore, overexpression of *GhCIPK6a* in Upland cotton improved salt tolerance in seed germination stage through increasing the stability of cell membrane.

**Fig. 8:**
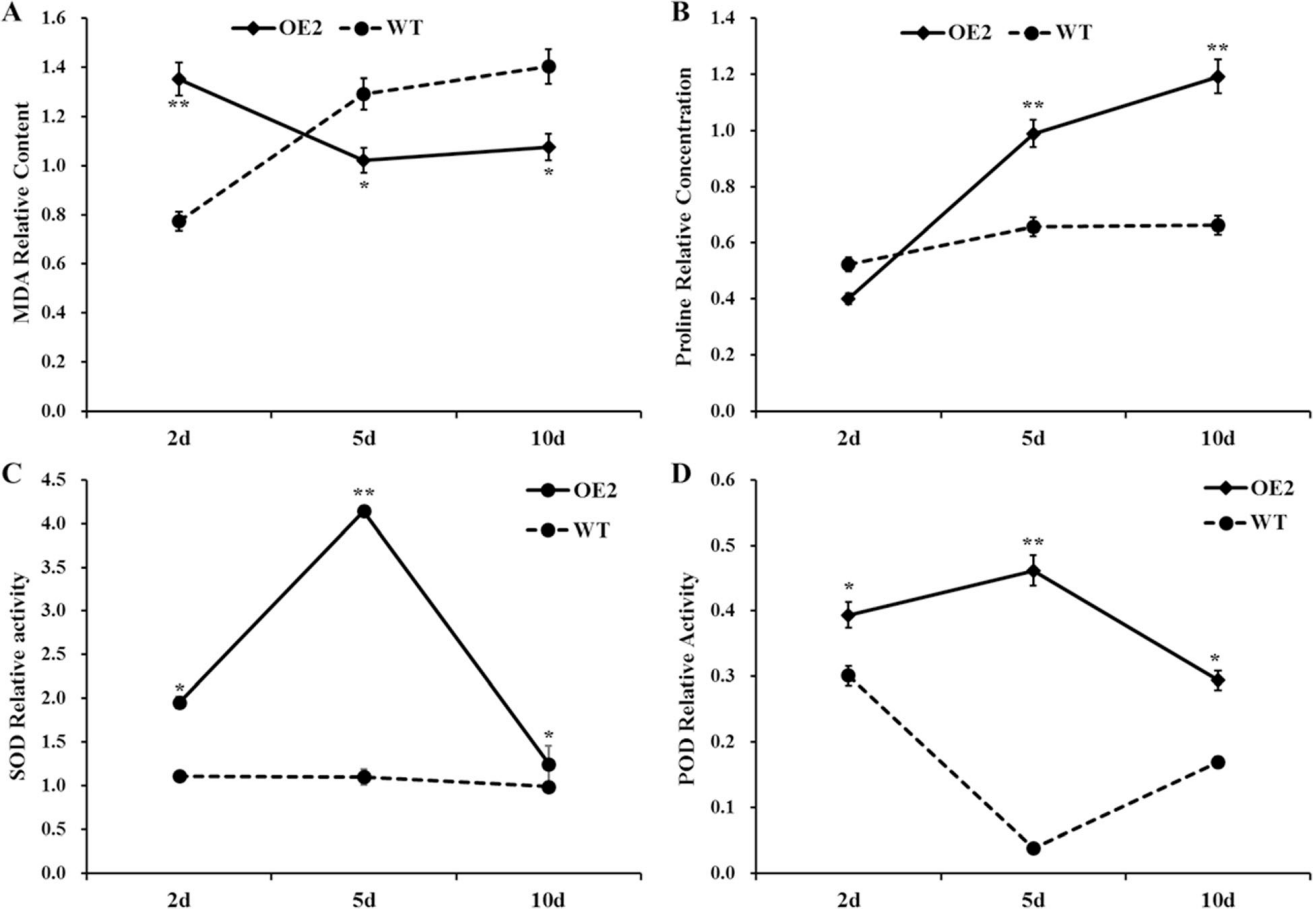
Statistical analysis of physiological indexes in leaves of transgenic (OE2) line and wild-type (WT) line seedlings under 150 mmol·L^− 1^ NaCl treatment. **a**. The relative content of MDA in the leaves of transgenic line (OE2) and wild-type (WT) line after 2, 5, and 10 days of 150 mmol·L^− 1^ NaCl treatment. **b**. The relative concentration of proline in the leaves of transgenic line (OE2) and wild-type (WT) line after 2, 5, and 10 days of 150 mmol·L^− 1^ NaCl treatment. **c** and **d**. The relative activity of SOD (**c**) and POD (**d**) in the leaves of transgenic line (OE2) and wild-type (WT) line after 2, 5, and 10 days of 150 mmol·L^− 1^ NaCl treatment. *, *p*-value < 0.05; **, *p*-value < 0.01

To confirm that overexpression of *GhCIPK6a* enhanced salt tolerance during seedling stage, we treated transgenic and wild-type seedlings at three-leaf stage by soaking the roots in hydroponic solution with 150 mmol·L^− 1^ NaCl. We determined the MDA and proline contents, and SOD and POD activities of seedlings exposed to salt stress. After 2, 5, and 10 d of salt treatment, the transgenic line maintained lower relative content of MDA than control, but relative content of proline and relative activities of POD and SOD were higher than those of wild-type (Fig. 8). Since MDA and proline promote membrane stability, and POD and SOD limit membrane lipid peroxidation by reducing the accumulation of H_2_O_2_. So the result suggests that over-expression of *GhCIPK6a* increases both the POD and SOD activities, and thereby reduces H_2_O_2_ accumulation and protects plant seedlings from membrane damage under salt stress*.*

### Improved salt tolerance of *GhCIPK6a* transgenic cotton in field experiment

To evaluate the salt tolerance of the *GhCIPK6a*-overexpressed cotton plants, we planted the T_6_ and T_7_ generation transgenic lines and the control under two different conditions in the field experiment in Handan City, Hebei Province, China. The generation processes of transgenic lines were shown in Additional file 8 Fig. S3c and S3d. Table 2 showed the yield and fiber quality traits of transgenic lines and wild type control in 2016 and 2017.

**Table 2:** Yield and fiber quality traits of *GhCIPK6a* transgenic lines and wild type under normal and saline conditions in 2016 and 2017

Condition	Generation	Line ID	Boll number	Boll weight (g)	Lint percentage (%)	Fiber length (mm)	Fiber uniformity rate (%)	Fiber strength (cN/tex)	Fiber elongation (%)	Micronaire
Normal	T_6_	OE1	14.7	5.3	38.0	30.4*	85.9*	29.9	6.9	5.2
		OE2	14.8	5.4	38.9*	30.2	86.0**	29.9	6.9	5.3*
		WT	14.7	5.5	37.0	29.9	85.1	29.6	6.9	5.2
	T_7_	OE1	23.6*	6.0	42.2	29.1	84.9	27.9	6.9	5.3
		OE2	23.7**	6.1	43.1	29.5	85.2	27.8	6.9	5.2
		WT	22.1	6.2	42.1	29.6	85.4	26.8	6.9	5.2
Saline	T_6_	OE1	15.1*	5.2	38.0*	29.9	86.5**	29.7	6.7	5.3
		OE2	15.9**	5.3	38.3**	30.2	86.0**	29.9	6.7	5.3
		WT	13.5	5.3	37.5	30.1	84.2	30.4	6.7	5.6
	T_7_	OE1	22.4	5.5	43.0	30.8	85.0	28.8	6.8	5.5
		OE2	22.1	5.7	43.3	30.7	85.3	28.5	6.7*	5.5
		WT	22.9	5.6	43.4	30.5	85.4	28.2	6.8	5.4

*, *p*-value < 0.05

**, *p*-value < 0.01

**Fig. 9:**
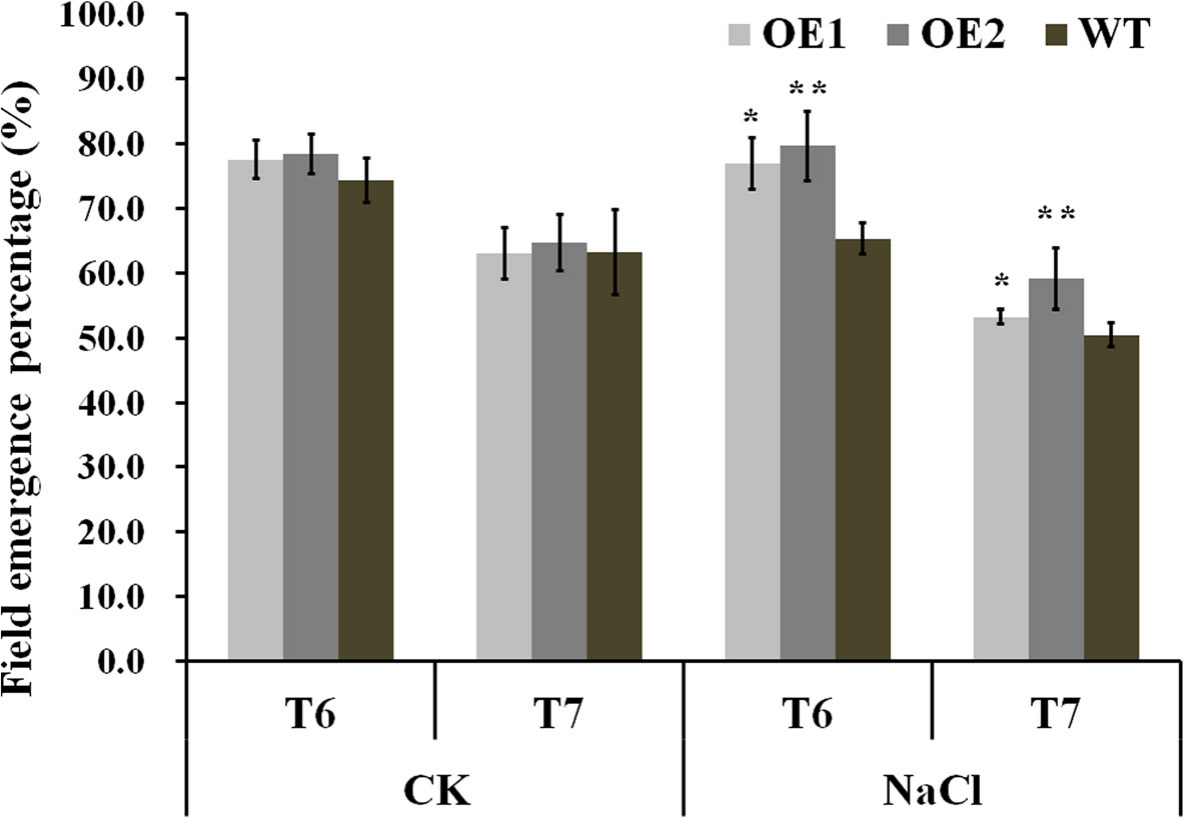
The seedling field emergence percentage of transgenic lines (OE1 and OE2) and wild-type (WT) line under normal and saline conditions in 2016 and 2017. *, *p*-value < 0.05; **, *p*-value < 0.01

During seedling stage, the seedling field emergence percentage of *GhCIPK6a* overexpression lines and WT line were similar under normal condition. Under salt stress, the seedling field emergence percentage of OE lines was significantly higher than WT lines over 2 years (Fig. 9). In the flowering and boll periods, there was a hail disaster on June 28, 2016, which caused the boll number of OEs and WT lines less than that in 2017 (Table 2). Meanwhile, the transgenic lines recovered better than the WT line after hail disaster, especially under salt stress. The stronger resilience of OE lines was shown in more boll number and higher lint percentage than that in WT line, under salt condition in 2016 (Table 2). Otherwise, the fiber uniformity rate of *GhCIPK6a* overexpressed lines was significantly higher than that in WT line, which indicated that *GhCIPK6a* overexpressed lines showed stronger adaptability and resilience in extreme environments.

In 2017, the boll number of transgenic lines was significantly higher than that in WT line under normal condition, which was no significant difference under salt stress. In addition, the boll weight and lint percentage were no significant differences between OE and WT lines. It can be speculated that *GhCIPK6a* overexpressed in Upland cotton could increase yield under normal condition. There was no influence in fiber quality trait. Under salt stress, there were also no differences in yield and fiber quality traits between overexpression and wild type lines (Table 2). Therefore*, GhCIPK6a* overexpressed in cotton increased the seedling field emergence percentage, seed cotton yield under salt condition, and maintained the stability of yield and fiber quality traits under extreme treatments.

We also evaluated the salt tolerance of transgenic and control cottons during flowering and boll setting stage in the field in Akesu City, Xinjiang Autonomous Region, China in 2013 (Additional file 11 Fig. S6). The salt content of the soil under the surface 5 to 10 cm was approximately 0.92%, which was significantly higher than the tolerance of cotton. Under severe salt stress, there were extensive necrosis in the leaves occurred in the wild type plants and ZG5, a salt sensitive cotton variety [41, 72]. However, the transgenic plants and Z9806 (a salt tolerant cotton variety [12]) could grow very well, and show no necrosis in leaves (Additional file 11 Fig. S6).

### *GhCIPK6a* involved in MAPK signaling pathway and plant hormone signal transduction pathway to response to salt stress

**Fig. 10:**
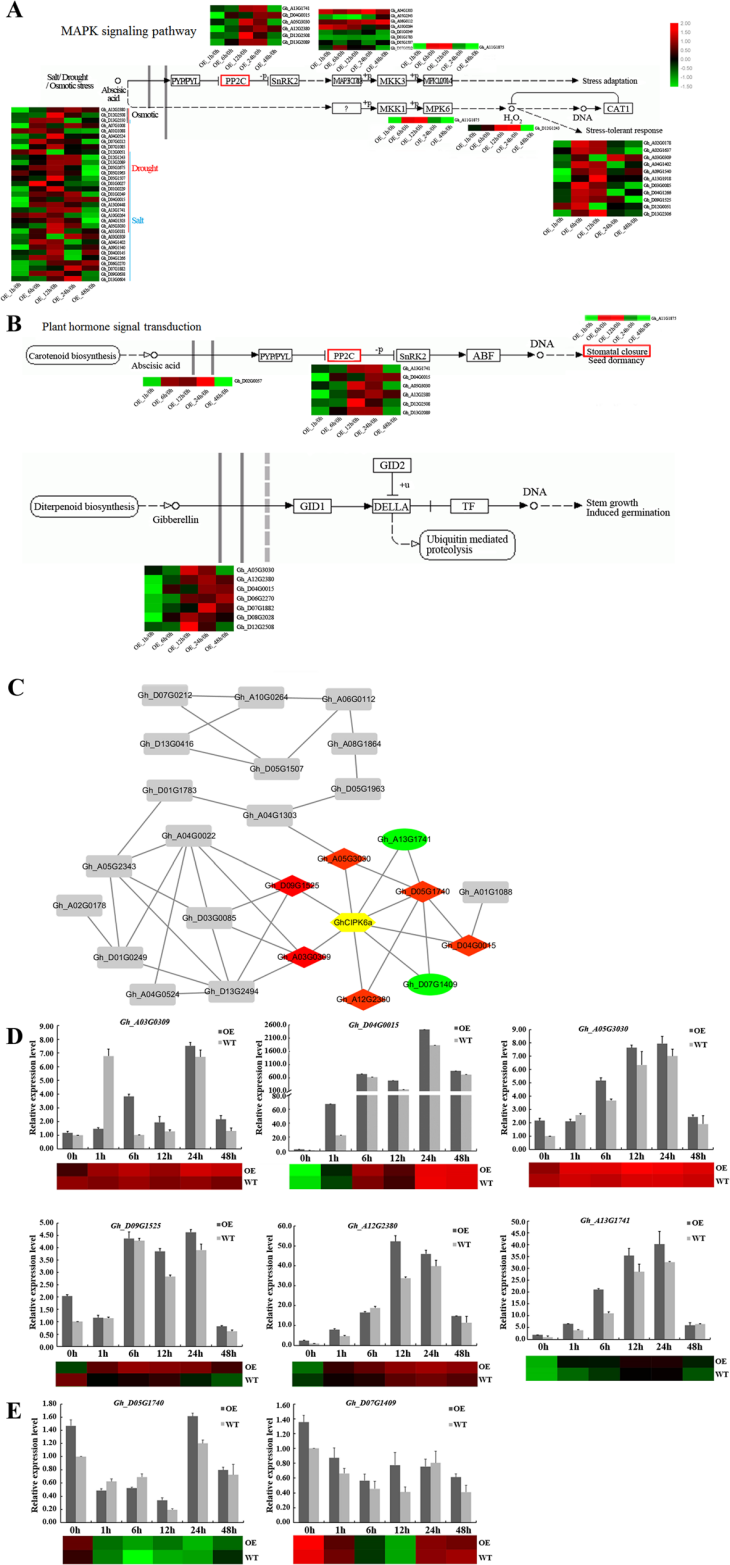
The specifically expressed DEGs in OE2 plants analysis. **a** and **b**. The DEGs enriched KEGG pathways and expression profile analysis. **c.** The PPI network predicted using STRING program. The genes with red rhombus background were co-expressed genes in up-regulated DEGs. The genes with green oval background were co-expressed genes from down-regulated DEGs. The genes with gray rectangular background were the ones predicted in the same PPI network with *GhCIPK6a* from up-regulated DEGs. **d.** Verification of expression profile of the up-regulated DEGs co-expressed with *GhCIPK6a*. **e.** Verification of expression profile of the down-regulated DEGs co-expressed with *GhCIPK6a*

In order to understand the mechanism of *GhCIPK6a* overexpressing to improve salt tolerance in transgenic cotton, we analyzed the transcriptome of *GhCIPK6a*-overespression (OE2) line and wild-type plants. A total of 252 genes were up- and 79 genes were down-regulated, respectively, in *GhCIPK6a-*overexpression compare to wild-type plants (Additional file 12 Fig. S7, Additional file 1 Table S1). In 252 up-regulated DEGs, GO-term analysis indicated 78 genes enriched in response to signaling, stress, ROS and stimulus progresses (Additional file 13 Fig. S8a, b). Among the 78 candidate genes, there were 33 genes response to salt, osmic, and drought stresses (Fig. 10a, b), eight genes involved in MAPK cascade (GO:0000165). The gene *Gh_A11G1875* predicted in activation of MAPKK activity process (GO:0000186) and regulation of stomatal closure (GO:0090333). There was one gene, *Gh_D02G0057*, response to ABA stimulus (GO:0071215), and seven genes were response to gibberellin acid (GO:0009739). KEGG pathways analysis showed the 78 genes mainly enriched in to signal transduction pathways, especially in MAPK signaling pathway (ko04016) and plant hormone signal transduction pathway (ko04075) (Fig. 10a, b, Additional file 13 Fig. S8c).

Protein interactions among DEGs was detected using the online STRING program, there were 23 genes co-expressed with *GhCIPK6a* within 78 up-regulated genes, and two genes co-expressed with *GhCIPK6a* in 79 down-regulated DEGs (Additional file 2 Table S2). Combined the information of edges and nodes of up- and down-regulated genes, the PPI network was conducted using Cytoscape software (Fig. 10c).

In PPI network, there were eight genes predicted to co-express with *GhCIPK6a* directly, six up- and two down-regulated DEGs. Six up-regulated DEGs were *Gh_A03G0309* (18.5 kD class I heat shock protein, HSP18.5-C), *Gh_A05G3030* (Protein phosphatase 2C 37, PP2CA), *Gh_A12G2380* (Probable protein phosphatase 2C 75, AHG1), *Gh_A13G1741* (Protein phosphatase 2C 56, ABI1), *Gh_D04G0015* (Probable protein phosphatase 2C 8, PP2C8), and *Gh_D09G1525* (18.2 kD class I heat shock protein, HSP18.2), respectively. All PP2C genes were involved in MAPK signaling pathway (ko04016) and plant hormone signal transduction pathways (ko04075) (Fig. 10c). Two *HSP* genes were involved in Protein processing in endoplasmic reticulum (ko04141). Two down-regulated DEGs were *Gh_D05G1740* (Serine/threonine-protein kinase STY46) and *Gh_D07G1409* (Probable serine/threonine-protein kinase), which were no KEGG annotation, both enriched in the MAPKKK activity process (GO:0004709). All DEGs in the PPI network were verified using qRT-PCR (Fig. 10d, e, Additional file 14 Fig. S9) and were consistent with the RNA-seq data.

### Overexpressed *GhCIPK6a* also improve the tolerance to osmotic and low-temperature stresses

To investigate the tolerance to abiotic stresses of transgenic cotton lines, we also determined seed germination potential and seed germination rate under drought (15% PEG 6000) and low-temperature stresses (distilled water, at 15 °C), respectively. Seed germination rate of transgenic cottons were significantly higher than wild type under drought (15% PEG 6000) and low-temperature stresses (Fig. 6a).

## Discussion

To develop the gene sources for molecular breeding is a popular strategy used in genetic improvement to improve abiotic stresses. Here, *GhCIPK6a* (HM002633) has the potential to improve the salt tolerance in cotton*. GhCIPK6a* shared 66.22% nucleotide sequence identity with *AtCIPK6*. *GhCIPK6a* expression was significantly increased by salt treatment (Additional file 7 Fig. S2). Overexpression of *GhCIPK6a* enhanced the seed germination rate under various stress treatments.

### The difference between GhCIPK6a (HM002633) and GhCIPK6 (KC465063)

Another GhCIPK6 gene (*GhCIPK6*(KC465063)) was previosly reported in Upland cotton [42, 44]. The *GhCIPK6a* (HM002633) identified here shared 78.56% nucleotide sequence identity with *GhCIPK6* (KC465063), and 90.49% amino acid sequence identity. We further analyzed the *GhCIPK6a* (HM002633) and *GhCIPK6* (KC465063) sequences in the A_2_, D_5_, and AD_1_ genomes (Table 1). The homolog IDs of *GhCIPK6a* (HM002633) in the A_2_, D_5_ and AD_1_ genomes are *Cotton_A_01247*, *Gorai.009G055400.1*, *Gh_A05G0418* and *Gh_D05G0536*, respectively. There are three homologs of *GhCIPK6* (KC465063)*, Gorai.010G112400.1* in the D_5_ genome, *Gh_A06G0873* and *Gh_D06G1020* in AD_1_ genome, but there was no homolog in the A_2_ genome. Each predicted chromosome locations of *GhCIPK6a* (HM002633) and *GhCIPK6* (KC465063) in A_2_, D_5_, and AD_1_ genomes were shown in Table 1. Multiple sequence alignment and phylogenic analysis confirmed that the two *GhCIPK6s* were two different genes in sequence. The gene *GhCIPK6* (KC465063) might be a paralog of *GhCIPK6a* (HM002633), created by cotton-specific duplication and evolution.

To confirm that the differences between *GhCIPK6a* (HM002633) and *GhCIPK6* (KC465063) not only involved their nucleotide sequences, we analyzed the phosphorylated sites in amino acid sequences (Additional file 3 Table S3, Additional file 15 Fig. S10). Differences between amino acid sequences caused differences in the phosphorylated sites that might activate downstream genes.

Using public data, we analyzed the relative expression levels of *GhCIPK6a* (HM002633) and *GhCIPK6* (KC465063) in different cotton tissues, at different stages of fiber development and subjected to various stresses (Additional file 9 Fig. S4). We found that the expression level of *GhCIPK6a* (HM002633) was strongly higher than *GhCIPK6* (KC465063) in different tissues under different treatments. *GhCIPK6a* (HM002633) was more sensitive to salt stress than *GhCIPK6* (KC465063). The transcripts of *GhCIPK6a* (HM002633) was up-regulated to a greater extent in roots of salt-tolerant and salt-sensitive cotton cultivars after different salt treatment times. Heterologous expression of *GhCIPK6* (KC465063) significantly enhanced tolerance to salt, drought, and ABA treatments in transgenic Arabidopsis, but there was no evidence that this gene could increase abiotic tolerance when overexpressed in cotton. Therefore, abiotic stress had a greater effect on *GhCIPK6a* (HM002633) expression than that on *GhCIPK6* (KC465063).

### GhCIPK6a can be used in cotton stress-tolerance breeding

Seed germination stage is the most sensitive stage to salt stress for cotton development [76], and the seed germination rate of over-expressing *GhCIPK6a* lines was higher than that of wild type under abiotic stress. The effect of salt stress on cell membrane permeability was detected by determining the electrical conductivity after soaking the seeds for 24 h in different concentrations of NaCl solution since cotton is particularly sensitive to abiotic stresses at the germination stage. Compared with control, the transgenic line showed higher seed germination rate under varied stresses. In imbibed germination stage, the water absorbency rate of transgenic cotton also maintained a normal and stable level under salt stress. After 24 h of soaking in NaCl solution, the cell membrane of transgenic line (OE2) was more stable than control (Figs. 6*,* 7b).

Seed germination indexes include root length and hypocotyl length, which were measured at nine days after germination under salt and control treatments. There was no significant difference between the transgenic and wild-type cotton in seedlings growth under salt treatment and normal conditions (data not shown). In the normal and saline fields, the growth performance during the seedling and flowering stages were investigated, between *GhCIPK6a* overexpression lines and wild type line. The growth of transgenic and wild-type cottons was inhibited by salt stress without significant difference between them (data not shown). These results suggest that overexpression of *GhCIPK6a* does not relieve the inhibitory effect to seedlings growth of salt stress.

Under intense salt stress, overexpressed *GhCIPK6a* cotton plants could grow better than wild type cotton (Additional file 11 Fig. S6). The transgenic lines, and receptors, with salt sensitive and resistive varieties were planted in the natural salinity field in Akesu, Xinjiang Autonomous Region, China in 2013, which soil salt content below the surface 5 to 10 cm was approximately 0.92%. The OE2 line could grow better than other varieties in the salinity field. The damage degree of salt stress of OE2 cotton was lighter than controls (Additional file 11 Fig. S6). Meanwhile, seed germination, growth, yield and fiber quality traits of transgenic cotton lines were not decreased compare to wild type cotton, especially overexpressed *GhCIPK6a* plants showed higher adaptive capacity than receptor plants under extreme environments (Fig. 9*,* Table 2).

### *GhCIPK6a* involved in multiple salt responsive pathways to response salt stress

In present study, we determined relative content of MDA in the shoots of transgenic and wild-type cotton under salt stress and normal conditions (Fig. 8). Under salt stress, relative MDA content in the OE2 line was maintained at around 1.00. However, in wild-type cotton, relative content of MDA increased with treatment time lasted. After 10 d exposure to salt stress, the MDA content of wild-type cotton was approximately 1.40-fold higher than control. The proline content in the OE2 and wild-type lines were both increased, but the increase was greater in OE2. Soil salt causes changes in physiological indexes while plants come across stress. Physiological indexes, including MDA, proline content, and the activity of antioxidant enzymes (POD and SOD), are typical parameters for evaluating abiotic stress tolerance. Plants with lower relative levels of MDA and higher levels of proline tend to maintain cell membrane stability under stresses [77]. Under salt treatment, the salt-tolerance cotton genotype displayed higher plant dry weight, photosynthesis and antioxidant enzymes activities [78]. Similarly, the activities of POD and SOD were higher in OE2 than wild type under salt stress, which implied that the transgenic cotton had a greater ability to scavenge the ROS in response to salt stress. Furthermore, the higher the activity of POD and SOD under stresses, the better the plant is able to scavenge over-produced ROS and protect its cells from ROS damage [16]. Among RNA-seq analysis results, there were some DEGs involved in the response to reactive oxygen species pathway (GO:0000302) in OE2 plants (Additional file 13 Fig. S8), which demonstrated *GhCIPK6a* overexpression could improve the express levels of peroxidase related genes to enhance ROS scavenging ability.

CIPKs are involved in a variety of stress responsive processes, function in multiple regulatory pathways, such as the SOS pathway, the Low-K^+^ response pathway [24, 74], and the ABA signaling pathway [79]. *GhPP2C* and *GhSnRK2.6* were both strongly induced in OE2 plants under salt stress. Also, in RNA-seq analysis, there were four PP2C genes predicted to co-express with *GhCIPK6a* in transgenic plants under salt treatment (Fig. 10*,* Additional file 2 Table S2). It was speculated that GhCIPK6a is involved in the MAPK signaling pathway and plant hormone signal transduction pathway, by co-expressed with GhPP2C proteins.

The CIPK family of protein kinases regulate ion homeostasis by forming a complex with CBL proteins. The CBL4/CIPK6 complex in Arabidopsis modulates the AKT2 potassium channel in the plasma membrane [80]. In vitro CBL1, CIPK23, and AKT1 can act together to mediate the CBL-dependent enhancement of phosphorylation of target proteins by CIPKs [81]. CIPKs modulate the activity of the genes encoding K^+^ channels to mediate root K^+^ uptake, such as AtAKT1 and AtAKT2 in Arabidopsis [24, 55, 80, 82]. In wild halophyte *Hordeum* *brevisubulatum*, HbCIPK2, combined with HbCBL1, could activate HbVGKC1 to absorb K^+^, combined with HbCBL4/10 could modulate HbSOS1L to exclude Na^+^ [83]. Overexpression of *GhCIPK6a* in cotton might enhance tolerance to abiotic stress by interacting with GhCBLs, GhPP2C, and GhSnRK2.6, also regulates peroxidase activity, which were also verified using RNA-seq analysis between OE2 and WT cottons (Fig. 10, Additional file 13 Fig. S8). Thus, *GhCIPK6a* probably functions in various signal pathways that mediate response to abiotic stresses in cotton. In conclusion, *GhCIPK6a* could function in multiple stress responsive pathways, such as K^+^ uptake pathway (Fig. 5), ROS scavenging pathway (Fig. 8, Additional file 13 Fig. S8a), MAPK signaling pathway and plant hormone signal transduction pathway (Fig. 10a, b), to enhance salt tolerance in transgenic cotton. In one word, this work shows that *GhCIPK6a* overexpression can improve the salt tolerance in cotton.

## Conclusions

We isolated the *GhCIPK6a* from Upland cotton under salt stress, and verified the function of *GhCIPK6a* by transformation and RNA-seq analysis. *GhCIPK6a* overexpressed lines exhibited higher seed germination rate than wild-type cotton under abiotic stresses, which functioned by involving in multiple stress responsive pathways, such as the K^+^ uptake pathway, the ROS scavenging pathway, MAPK signaling pathway and plant hormone signal transduction pathway. Moreover, *GhCIPK6a* overexpressed lines increased lint percentage, and fiber length uniformity under salt stress in the field. Therefore, *GhCIPK6a* has the potential for cotton breeding to improve stress-tolerance.

## Methods

### Plant materials and plant growth conditions

The Upland cotton cultivar ‘11–0516’ (a line bred from CCRI 12 in our laboratory) was used for genetic transformation. To amplify candidate gene sequences, cDNA and genomic DNA were isolated from the Upland cotton cultivar ‘Zhong G5’ [41, 72]. The ‘Zhong G5’seeds were provided by Chinese Academy of Agricultural Sciences primitively. The cultivars ‘11-0516’ and ‘Zhong G5’ are conserved by our laboratory now.

Upland cotton seedlings were grown in modified 1/2 Hoagland solution. Plants were grown at 28/20 °C, a light intensity of 600 mol·cm^2^∙s^− 1^, and a photoperiod of 14 h light/10 h dark [41]. At the three-leaf stage, half of the seedlings were transferred to plates containing 150 mmol·L^− 1^ NaCl solution in the growth medium; the remaining seedlings was grown in normal nutrient solution as control. Roots, stems, and leaves of the treated seedlings and control ones were sampled separately at 1, 2, 3, 6, and 12 h after exposure to salt stress, immediately frozen with liquid nitrogen and stored at − 80 °C for RNA extraction and qRT-PCR analysis.

Total RNA was extracted, qualified, and quantified, contaminating genomic DNA was digested with DNase I, and the RNA was reverse transcribed into cDNA following a previously described method [41].

### Isolation and bioinformatics analysis of full-length *GhCIPK6a*

The primers which were designed using Primer Premier 5 software (PREMIER Biosoft International) and shown in Additional file 4 Table S4, were used to amplify the full-length cDNA of a previous reported CBL-interacting protein kinase (GW691274) from Upland cotton roots [41]. The fragment was obtained using a TaKaRa PCR Thermal Cycler Dice (TaKaRa Bio Inc.). The amplification conditions were as follows: 94 °C for 5 min, then 30 cycles at 94 °C for 30 s, 55 °C for 30 s, and 72 °C for 2 min, followed by 72 °C for 10 min. Then the fragment was purified and sequenced.

The amplified cDNA sequences were analyzed using Lasergene software (DNAStar, MD, USA). The gene structure was predicted using GSDS (Gene Structure Display Server, http://gsds.cbi.pku.edu.cn/) [73]. Protein conserved domains were predicted using ScanProsite online software (http://au.expasy.org/tools/scanprosite/).

Multiple sequence alignment of GhCIPK6a with CIPK proteins of other species was performed using the ClustalW program implemented in DNAMAN software, and the phylogenetic tree was constructed using MEGA5.2 software with the neighbor-joining method and the 1000 bootstrap test replicates.

### Subcellular localization of GhCIPK6a

To make GFP-tagged GhCIPK6a, the coding region of *GhCIPK6a* was amplified with primers containing *Kpn*I and *BamH*I restriction sites, and the PCR product was first cloned into pMD 18-T vector (TaKaRa), and then sequenced. After *Kpn*I and *BamH*I digestion, the PCR fragment was cloned into the p3301-GFP plasmid, resulting in p3301-GhCIPK6a: GFP.

For subcellular localization in onion (*Allium cepa*) epidermal cells, the fusion construct p3301-GhCIPK6a: GFP was transformed into onion epidermal cells. The p3301-GFP vector was also introduced as control. The protocols of transformation and observation were referred as previously described [10]. The onion epidermal cells were plasmolyzed in 30% sucrose solution.

### BiFC analysis of interaction between GhCIPK6a and GhCBLs

The fusion vectors pUC-GhCIPK6a-YFP^N^ and pUC-GhCBLs-YFP^C^ were constructed using gene-specific primers with restriction sites (Additional file 4 Table S4). The constructs were transiently co-expressed in onion epidermal cells [10]. After incubation for 22–24 h at 28 °C, YFP fluorescence in the transformed cells was observed by confocal laser scanning microscopy (Nikon, EZ C1).

### Overexpression vector construction and cotton transformation

The coding sequence of *GhCIPK6a* was amplified by PCR using LA-Taq DNA polymerase and gene-specific primers containing restriction enzyme sites (Additional file 4 Table S4). *GhCIPK6a* was cloned and inserted into the *Xba*I-*Sac*I site of the pBI121 vector, under the control of the CaMV 35S promoter. The recombinant plasmid was named pBI-GhCIPK6a. The construct was transferred into cotton cultivar (11–0516) using the pollen tube-pathway method [84]. Seedlings of independent lines of *GhCIPK6a* transgenic plants were identified using 5 g L^− 1^ kanamycin sulfate solution, and were further confirmed by amplification of *NPT*II, which primers were shown in Additional file 4 Table S4. The copy number of the inserted fragment was determined by Southern Blotting analysis.

### Physiological index determination

To investigate the tolerance of transgenic cotton lines to abiotic stress, germination assays were performed using filter paper roll upright method. Thirty seeds of each transgenic or control cotton line were placed in a roll of filter paper, supplemented with distilled water (Control), NaCl solution (150 mmol·L^− 1^) and PEG6000 solution (15%, w/w), respectively, and maintained at 28 °C for 9 days, three replicates. The seed germination rate was investigated on the ninth day. The seed germination rate = the number of germinated seeds at the ninth day / the total number of seeds × 100%. Root length and hypocotyl length of seedlings were measured on the ninth day. In addition, to evaluate the chilling tolerance of the transgenic lines, seeds were placed at 15 °C for 15 d and the seed germination rate was obtained, three replicates. To determine the effect of salt stress on germination stage, water absorptivity rates were measured during the imbibition stage. Twenty seeds of each transgenic or control line exposed to solution containing different NaCl concentrations (0, 100, 150, and 200 mmol·L^− 1^), and maintained at 28 °C, four replicates. The weight of seeds was determined each 2 h. The water absorbency rate = (the weight of seeds after water absorption – the initial weight of seeds before soaking) / the initial weight of seeds before soaking × 100%.

For seedling growth assay, transgenic seedlings and control were grown in modified 1/2 Hoagland solution. At the three-leaf stage, transgenic and control cotton seedlings were transferred to 1/2 Hoagland solution or solution supplemented with 150 mmol·L^− 1^ NaCl. Roots of salt-stressed and control seedlings were sampled at 1, 2, 3, 6, and 12 h after treatment, immediately frozen in liquid nitrogen, and then stored at − 80 °C until used for RNA extraction and qRT-PCR analysis. After growing for 2, 5, and 10 days under salt stress, leaves were sampled for determination of physiological indexes, including the MDA and proline contents, and antioxidant enzyme (SOD and POD) activity, followed previous methods [85–87]. All assays include three biological replications with six plants each replication and error bars indicate the standard deviation (SD).

### Field phenotype assay

To evaluate the salt tolerance of transgenic cotton lines during the whole growth and development period, field trials were carried out in Quzhou Experimental Station of China Agricultural University at Handan City (36°78′N, 114°92′E), Hebei Province, China, in summer season of 2016 to 2017. Cotton plants were planted in four row plots with 13 holes each row. Plots were 4 m in length with 80 cm row spacing for the experiment. Plants were spaced 33 cm in rows. Each hole planted eight seeds. The field design followed a randomized complete block design with four replications, respectively. Field management followed the local conventional standard field practices. The saline field was irrigated with 0.4% saline water before sowing, and the control field was irrigated with fresh water [12]. The receptor cultivar ‘11–0516’ was used as control in normal and salinity conditions.

The seedling field emergence percentage was investigated after sowing 30 days, which were used to reflect the status of the seedling emergency and survival. The seedling emergency percentage = the number of survival seedling / the number of total sowing seeds (eight seeds per hole× 13 holes per row × four rows) × 100%. Boll number was surveyed from 44 individuals in each plot on Sept 15th. Yield related traits consisted of boll weight (g) and lint percent (%). Fiber length (mm), fiber length uniformity (%), fiber strength (cN·tex^− 1^), fiber elongation (%), and Micronaire were determined with HVI 1000 (Uster® HVISPECTRUM, Spinlab, USA) by the Cotton Quality Supervision, Inspection and Testing Center, Ministry of Agriculture, Anyang, Henan Province, China.

### Expression analysis based on public expression data of cotton

Public cotton expression data (Additional file 5 Table S5) were obtained from PLEXdb (http://www.plexdb.org/index.php) and Gene expression Omnibus (GEO, http://www.ncbi.nlm.nih.gov/geo/). Transcriptome differences between the root tissues of two cotton cultivars (salt-tolerant ‘Zhong 07’ and salt-sensitive ‘Zhong G5’) were evaluated after 3, 12, and 48 h of exposure to salt stress [72], and expression profiles of candidate genes were also analyzed. All microarray data was normalized using robust multichip analysis (RMA). The expression profiles of several candidate genes were examined using quantitative RT-PCR in transgenic cotton plants exposed to salt stress. The heatmap was constructed by Mev software (http://mev.tm4.org/).

### RNA sequencing and analysis

Total RNA was extracted from the root samples of OE2 transgenic line and the receptor cultivar ‘11–0516’ seedlings, which were treated for 0, 1, 6, 12, 24 and 48 h, respectively, and three biological replicates. RNA-seq libraries were prepared and sequenced on an Illumina Hiseq2500 platform (Biomarker Technology Corporation, Beijing, China). All raw data were submitted in the NCBI sequence read archive (http://www.ncbi.nlm.nih.gov/sra) with project ID PRJNA644135 (https://www.ncbi.nlm.nih.gov/bioproject/PRJNA644135).

Significant differential expression analysis was using the DEGSeq method [88], defined as the fold change (salt treated cv 0 h) > 2 and FDR < 0.01, that analysis was performed using BMKCloud (www.biocloud.net). Gene Ontology (GO) analysis was conducted by WEGO online software. The DEGs enriched pathways analysis was used Kyoto Encyclopedia of Genes and Genomes (KEGG) databases. Protein–protein interaction (PPI) networks was established using the Search Tool for the Retrieval of Interacting Genes/Proteins (STRING) v11 (http://string-db.org/) and Cytoscape app (http://apps.cytoscape.org/apps/stringapp) [89, 90]. A combined score ≥ 0.4 was chosen for PPI network construction.

### Quantitative RT-PCR analysis

Quantitative RT-PCR analyzed the expression profiles of candidate genes in transgenic cotton plants and wild type subjected to salt stress. Quantitative RT-PCR was performed in triplicate with an ABI PRISM® 7500 Real-Time PCR System using the SYBR® Premix Ex Taq™ Kit (TaKaRa, DRR041A), according to the manufacturer’s instruction. The gene-specific primers were shown in Additional file 4 Table S4. The *GhUBQ7* transcript was used as a normalization control to quantify relative levels. Relative expression levels were calculated using the 2^-∆∆t^ method [91, 92]. Standard deviation was calculated from three biological replicates.

### Statistical analysis

Statistical analyses were performed using SPSS software (13.0 for Windows Evaluation Version, SPSS Inc., LEAD Technologies, USA). All significant differences were identified using a t-test and marked as *, *p*-value < 0.05; **, *p*-value < 0.01.

## Supplementary information

**Additional file 1: Table S1.** Up- and down-regulated DEGs in OE2 cotton treated by salt stress screening from RNA-seq analysis.

**Additional file 2: Table S2.** The edges and nodes information of Co-expression genes with *GhCIPK6a* in PPI network predicted using STRING online program.

**Additional file 3: Table S3.** Prediction of phosphorylated sites of GhCIPK6a (HM002633) and GhCIPK6 (KC465063) by KinasePhos (http://kinasephos.mbc.nctu.edu.tw/).

**Additional file 4: Table S4.** Primers used in vectors constructed and qRT-PCR. The underlined sequences were the restriction enzymes sequences.

**Additional file 5: Table S5.** Public cotton expression data from PLEXdb (http://www.plexdb.org/index.php) and the Gene expression Omnibus (GEO, http://www.ncbi.nlm.nih.gov/geo/).

**Additional file 6: Figure S1.** Phylogenic analysis of GhCIPK6a (HM002633) and CIPK homolog proteins from other species. Full-length amino acid sequences were aligned using the integrated ClustalW and phylogenetic tree was constructed using the neighbor-joining method implemented in MEGA5.2 (1000 bootstrap test replicates). Black triangles indicated GhCIPK6a (HM002633) and homologs from the A_2_, D_5_, and AD_1_ genomes, respectively. Black circles indicated GhCIPK6 (KC465063), and homologs from the D_5_ and AD_1_ genomes, respectively. The percentage of replicate trees was shown at the branches. Different colored branches represented different groups. Proteins, located in the branch marked by a blue triangle, were chosen for multiple alignments using DNAMAN software.

**Additional file 7: Figure S2.** Expression analysis of *GhCIPK6a* in different tissues after salt treatment. A. Expression analysis in Upland cotton cultivar ‘Zhong G5’; B. Expression profile in OE2 and wild-type lines.

**Additional file 8: Figure S3.** PCR analysis and Southern blotting assay of positive transgenic plants. A. Identification of positive T_1_ transgenic plants by amplifying the resistant gene *NPT*II. The samples marked in the red box were the positive T_1_ *GhCIPK6a* transgenic plants. M, D2000 DNA ladder; 1–11, positive individuals: 11 J100–1, 11 J100–2, 11 J100–7, 11 J100–8, 11 J100–12, 11 J100–15, 11 J100–21, 11 J100–24, 11 J100–27, 11 J100–34, 11 J100–35, respectively. CK+: positive control. The samples out of the red box were not involved here. B. Southern blotting assay of transgenic cotton plants using the resistance gene *NPT*II as the probe. In the red box: M, D2000 DNA ladder; 1, OE1 (12D44); 2, OE2 (12D47), which were the sample used in present study. 3–8, represents different individuals, which were 12D48-12D53, respectively. C and D. The genealogical diagram of OE1 (12D44) and OE2 (12D47) lines and their offspring.

**Additional file 9: Figure S4.** Expression profiles of *GhCIPK6s* under abiotic stress using public datasets. I, public data from PLEXdb. II, GSE50770, analyzed the crosstalk between genes that are responsive to multiple abiotic stresses, including ABA, cold, drought, salinity, and alkalinity (pH) in *G. hirsutum*. III, Transcriptome analysis between roots of the salt-tolerant cultivar ‘Zhong 07’ and salt-sensitive cultivar ‘Zhong G5’ after 3, 12, and 48 h of 150 mmol·L^− 1^ NaCl treatment.

**Additional file 10: Figure S5.** The germination performance of transgenic lines (OE1 and OE2) and wild type (WT) line under salt treatment (NaCl) and control (CK) conditions. Bar = 1 cm.

**Additional file 11: Figure S6.** Salt tolerance assay of transgenic and control cottons during flowering and boll setting stage in the field in Akesu, Xinjiang Autonomous Region, China in 2013. The salt content of the soil under the surface 5 to 10 cm was approximately 0.92%.

**Additional file 12: Figure S7.** Venn diagrams of up- and down-regulated DEGs among different salt stress time points in between OE2 and WT plants, identified from RNA-seq analysis.

**Additional file 13: Figure S8.** GO-term and KEGG analysis of up- and down-regulated DEGs. A. GO-term analysis of specific up- and down-regulated DEGs in OE plants following salt treatment. B. KEGG pathway analysis of 78 candidate DEGs.

**Additional file 14: Figure S9.** Validation of express profiles of candidate DEGs by qRT-PCR. A. RNA-seq analysis and qRT-PCR analysis of the co-expressed DEGs in PPI network. B. Correlation of the expression profiles between RNA-seq and qRT-PCR analysis.

**Additional file 15: Figure S10.** Analysis of differences in amino acid sequences and phosphorylation sites between GhCIPK6a (HM002633) and GhCIPK6 (KC465063). A. Sequence alignment of GhCIPK6a (HM002633) and GhCIPK6 (KC465063). B. Schematic of predicted phosphorylation sites of GhCIPK6s by KinasePhos (http://kinasephos.mbc.nctu.edu.tw/).

## Data Availability

All transcriptome raw data are available at NCBI project ID PRJNA644135 (https://www.ncbi.nlm.nih.gov/bioproject/PRJNA644135).

## Abbreviations

ABA: Abscisic acid
ABI: Abscisic acid-insensitive
BiFC: Bimolecular fluorescence complementation
CaMV: Cauliflower mosaic virus
CBL: Calcineurin B-like proteins
CIPK: CBL-interacting protein kinases
DEG: Differentially expressed genes
GEO: Gene expression Omnibus
GFP: Green fluorescent protein
GO: Gene Ontology
KEGG: Kyoto Encyclopedia of Genes and Genomes
MAPK: Mitogen-activated protein kinases
MDA: Dicarboxylic aldehyde
OE: Overexpression
PLEXdb: Plant Expression Database
POD: Peroxidase
PP2C: Protein phosphatase 2C
PPI: Protein phosphatase interaction
qRT-PCR: Quantitative real-time PCR
ROS: Reactive oxygen species
SD: Standard deviation
SnRK: Sucrose-nonfermenting-related kinases
SOD: Superoxide dismutase
SOS: Salt overly sensitive
WT: Wild type
