# Supplementary material for: GhCIPK6a increases salt tolerance in transgenic upland cotton by involving in ROS scavenging and MAPK signaling pathways
Source: BMC Plant Biol. 2020 Sep 14;20:421. doi: 10.1186/s12870-020-02548-4 (PMC7488661; doi:10.1186/s12870-020-02548-4)
Supplement: Supplementary file 3 — Additional file 3: Table S3. Prediction of phosphorylated sites of GhCIPK6a (HM002633) and GhCIPK6 (KC465063) by KinasePhos (http://kinasephos.mbc.nctu.edu.tw/). [file 12870_2020_2548_MOESM3_ESM.doc]

Additional file 3 Table S3. Prediction of phosphorylated sites of GhCIPK6a (HM002633) and GhCIPK6 (KC465063) by KinasePhos (http://kinasephos.mbc.nctu.edu.tw/).

| GhCIPK6a (HM002633) | | | GhCIPK6 (KC465063) | | |
| --- | --- | --- | --- | --- | --- |
| Locations (AA) | Phosphorylated Sites | Predictive Models | Locations (AA) | Phosphorylated Sites | Predictive Models |
| 67 | **KREISVMKM** | **[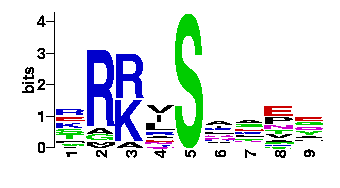](http://kinasephos.mbc.nctu.edu.tw/models2/S/PKG/PhosphoBase_PKG_S_4.txt.png)** | 67 | **KREISVMKM** | **[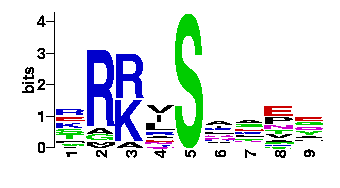](http://kinasephos.mbc.nctu.edu.tw/models2/S/PKG/PhosphoBase_PKG_S_4.txt.png)** |
| 91 | **KSKIYFAME** | **[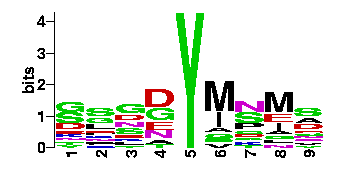](http://kinasephos.mbc.nctu.edu.tw/models2/Y/INSR/Mix_INSR_Y_4.txt.png)** | 91 | **KSKIYFAME** | **[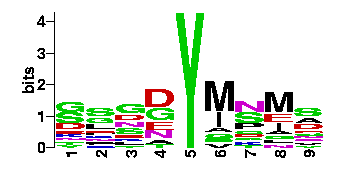](http://kinasephos.mbc.nctu.edu.tw/models2/Y/INSR/Mix_INSR_Y_4.txt.png)** |
| -- | **--** | -- | 179 | **TTCGTPAYV** | **[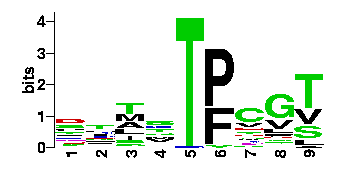](http://kinasephos.mbc.nctu.edu.tw/models2/T/Other_MDD/Mix_mdd_T_4.txt.grp.1.png)** |
| 182 | **GTSAYVAPE** | **[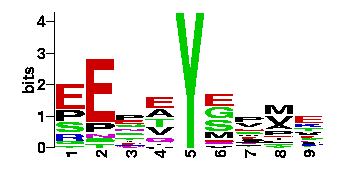](http://kinasephos.mbc.nctu.edu.tw/models2/Y/Src/PhosphoBase_SRC_Y_4.txt.png)** | 182 | **GTPAYVAPE** | **[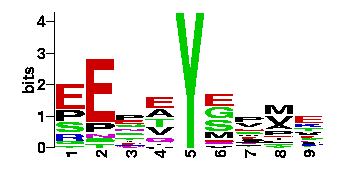](http://kinasephos.mbc.nctu.edu.tw/models2/Y/Src/PhosphoBase_SRC_Y_4.txt.png)** |
| 193 | **GKKGYDGAK** | **[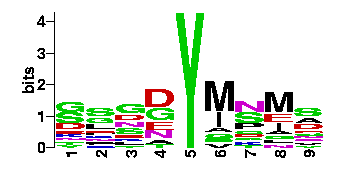](http://kinasephos.mbc.nctu.edu.tw/models2/Y/INSR/Mix_INSR_Y_4.txt.png)** | 193 | **GKKGYDGAK** | **[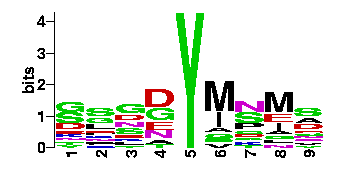](http://kinasephos.mbc.nctu.edu.tw/models2/Y/INSR/Mix_INSR_Y_4.txt.png)** |
| -- | **--** | -- | 263 | **RIAISKITE** | **[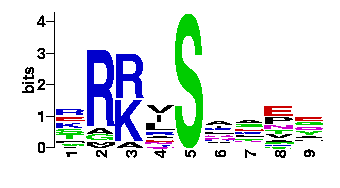](http://kinasephos.mbc.nctu.edu.tw/models2/S/PKG/PhosphoBase_PKG_S_4.txt.png)** |
| 355 | **SMNFSVKKS** | **[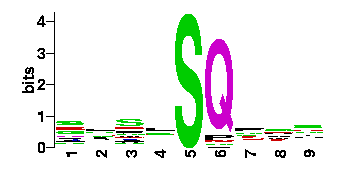](http://kinasephos.mbc.nctu.edu.tw/models2/S/ATM/Mix_ATM_S_4.txt.png)** | 355 | **SGKFSVKKS** | **[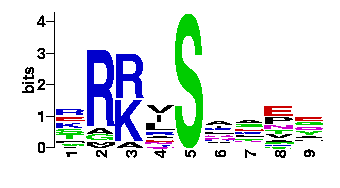](http://kinasephos.mbc.nctu.edu.tw/models2/S/PKG/PhosphoBase_PKG_S_4.txt.png)** |
| 359 | **SVKKSESSV** | **[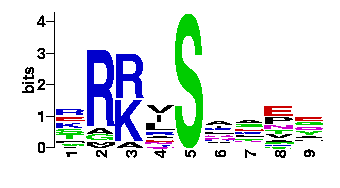](http://kinasephos.mbc.nctu.edu.tw/models2/S/PKG/PhosphoBase_PKG_S_4.txt.png)** | -- | -- | -- |
| 362 | **KSESSVRLQ** | **[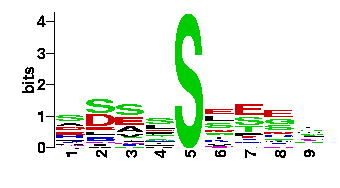](http://kinasephos.mbc.nctu.edu.tw/models2/S/CKI/Mix_CK1_S_4.txt.png)** | -- | -- | -- |

**S**, Serine; **T**, Threonine; **Y**, Tyrosine; --, no data.
