# Supplementary material for: GhCIPK6a increases salt tolerance in transgenic upland cotton by involving in ROS scavenging and MAPK signaling pathways
Source: BMC Plant Biol. 2020 Sep 14;20:421. doi: 10.1186/s12870-020-02548-4 (PMC7488661; doi:10.1186/s12870-020-02548-4)
Supplement: Supplementary file 5 — Additional file 5: Table S5. Public cotton expression data from PLEXdb (http://www.plexdb.org/index.php) and the Gene expression Omnibus (GEO, http://www.ncbi.nlm.nih.gov/geo/). [file 12870_2020_2548_MOESM5_ESM.doc]

Additional file 5 Table S5. Public cotton expression datasets from PLEXdb (<http://www.plexdb.org/index.php>) and the Gene expression Omnibus (GEO, <http://www.ncbi.nlm.nih.gov/geo/>).

| GEO Accession | Experiment Name | Stress condition | Tissue type name | References |
| --- | --- | --- | --- | --- |
| GSE16467 | GO1: Global gene expression compared between leaves of cotton plants (variety Sicot 71) flooded for 24 h and not flooded. | Flooded, control | Root, leaf | Christianson et al. 2010 |
| GSE18253 | GO2: Microarray data from field-grown drought-stressed *G. hirsutum* leaf. | Control, drought | Leaf | Cottee et al. 2014 |
| GSE29566 | GO7: Global gene expression analysis in the leaf tissue of cotton (*Gossypium hirsutum* L.) under drought stress. | Control, drought | Leaf | Paterson et al. 2012 |
| GSE29567 | GO8: Microarray analysis of global gene expression in cotton during fiber development (0, 5, 10, and 20 dpa^*^) under drought stress and field conditions. | Control, drought | Fiber development stages (0, 5, 10, and 20 dpa) | Padmalatha et al. 2012 |
| GSE29810 | GO9: Microarray analysis of *G. hirsutum* under drought stress in leaf tissue and during fiber development (stages 0, 5, 10, 15, and 20 dpa). | Control, drought | Leaf tissue; fiber development stages (0, 5, 10, and 20 dpa) | Nigam and Sawant 2013 |
| GSE50770 | Microarrays used to analyze the crosstalk between genes that respond to multiple abiotic stresses in cotton (*Gossypium hirsutum* L.). | ABA, cold, drought, salinity and alkalinity | Seedlings of 14-day after germination | Zhu et al. 2013 |

^*^dpa, day post anthesis
