# Supplementary material for: GhCIPK6a increases salt tolerance in transgenic upland cotton by involving in ROS scavenging and MAPK signaling pathways
Source: BMC Plant Biol. 2020 Sep 14;20:421. doi: 10.1186/s12870-020-02548-4 (PMC7488661; doi:10.1186/s12870-020-02548-4)
Supplement: Supplementary file 6 — Additional file 6: Figure S1. Phylogenic analysis of GhCIPK6a (HM002633) and CIPK homolog proteins from other species. Full-length amino acid sequences were aligned using the integrated ClustalW and phylogenetic tree was constructed using the neighbor-joining method implemented in MEGA5.2 (1000 bootstrap test replicates). Black triangles indicated GhCIPK6a (HM002633) and homologs from the A2, D5, and AD1 genomes, respectively. Black circles indicated GhCIPK6 (KC465063), and homologs from the D5 and AD1 genomes, respectively. The percentage of replicate trees was shown at the branches. Different colored branches represented different groups. Proteins, located in the branch marked by a blue triangle, were chosen for multiple alignments using DNAMAN software. [file 12870_2020_2548_MOESM6_ESM.docx]

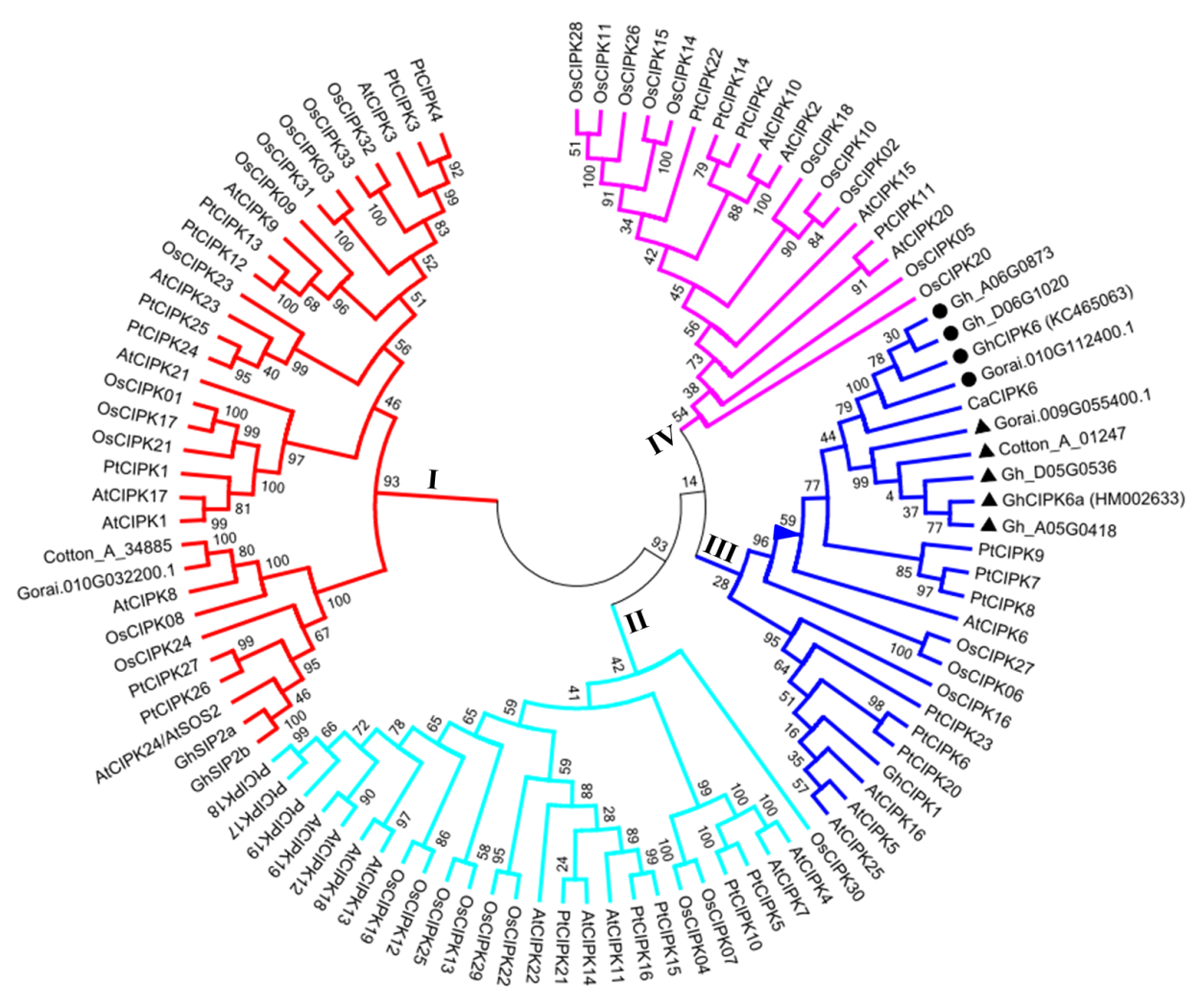


**Additional file 6 Figure S1.** Phylogenic analysis of GhCIPK6a and CIPK homolog proteins from other species. Full-length amino acid sequences were aligned using the integrated ClustalW and phylogenetic tree was constructed using the neighbor-joining method implemented in MEGA5.2 (1,000 bootstrap test replicates). Black triangles indicated GhCIPK6a (HM002633) and homologs from the A_2_, D_5_, and AD_1_ genomes, respectively. Black circles indicated GhCIPK6 (KC465063), and homologs from the D_5_ and AD_1_ genomes, respectively. The percentage of replicate trees was shown at the branches. Different colored branches represented different groups. Proteins, located in the branch marked by a blue triangle, were chosen for multiple alignments using DNAMAN software. At, *Arabidopsis thaliana*; Ca, *Cicer arietinum*; Pt, *Populus trichocarpa*; Os, *Oryza sativa*; Gh, *G*. *hirsutum* (AD_1_); Cotton_A, *G*. *arboreum* (A_2_); Gorai, *G*. *raimondii* (D_5_).
