# Supplementary material for: GhCIPK6a increases salt tolerance in transgenic upland cotton by involving in ROS scavenging and MAPK signaling pathways
Source: BMC Plant Biol. 2020 Sep 14;20:421. doi: 10.1186/s12870-020-02548-4 (PMC7488661; doi:10.1186/s12870-020-02548-4)
Supplement: Supplementary file 7 — Additional file 7: Figure S2. Expression analysis of GhCIPK6a in different tissues after salt treatment. A. Expression analysis in Upland cotton cultivar ‘Zhong G5’; B. Expression profile in OE2 and wild-type lines. [file 12870_2020_2548_MOESM7_ESM.docx]

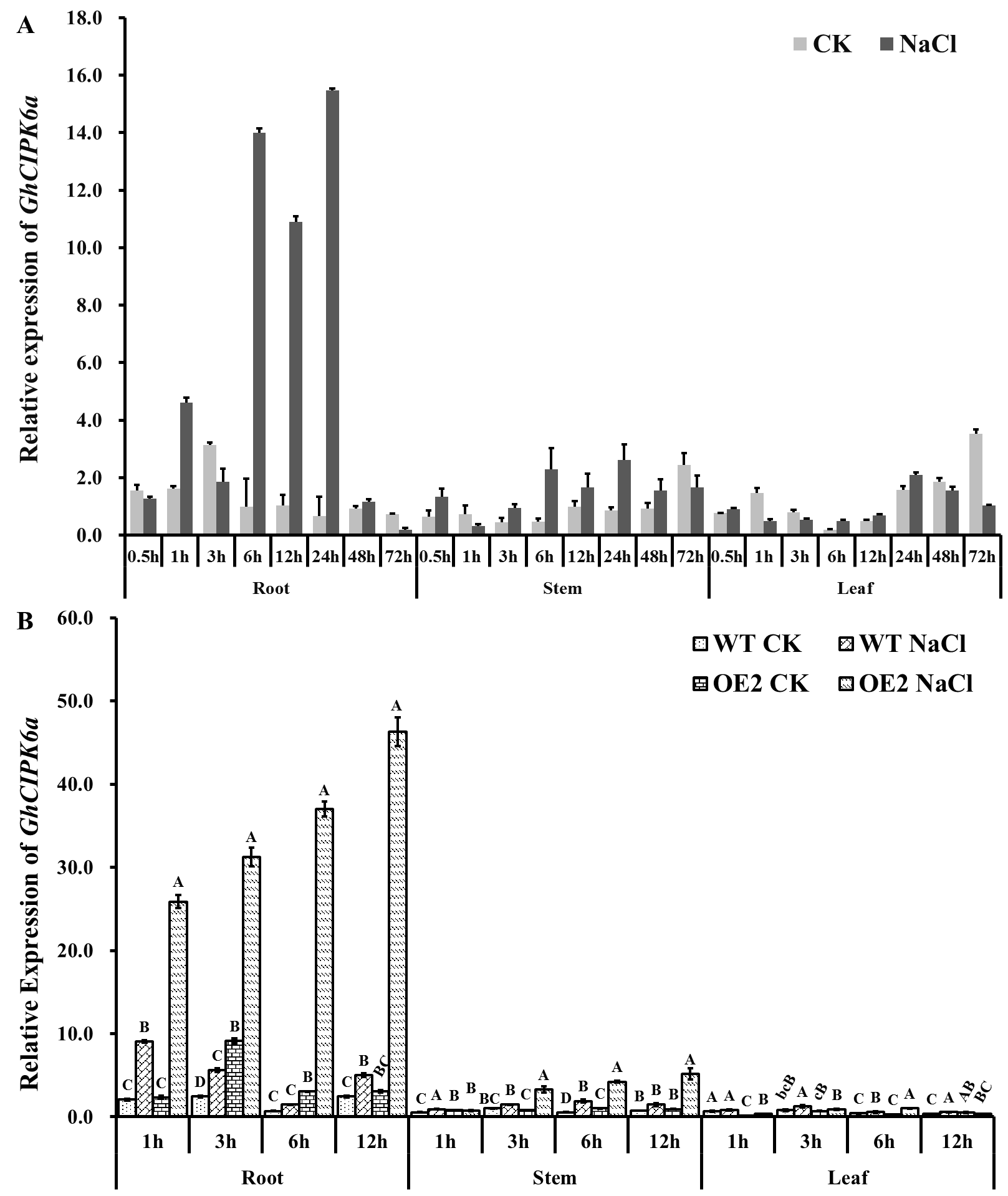


**Additional file 7 Figure S2.** Expression analysis of *GhCIPK6a* in different tissues after salt treatment. **A.** Expression analysis in Upland cotton cultivar ‘Zhong G5’; **B.** Expression profile in OE2 and wild-type lines.
