# Supplementary material for: GhCIPK6a increases salt tolerance in transgenic upland cotton by involving in ROS scavenging and MAPK signaling pathways
Source: BMC Plant Biol. 2020 Sep 14;20:421. doi: 10.1186/s12870-020-02548-4 (PMC7488661; doi:10.1186/s12870-020-02548-4)
Supplement: Supplementary file 8 — Additional file 8: Figure S3. PCR analysis and Southern blotting assay of positive transgenic plants. A. Identification of positive T1 transgenic plants by amplifying the resistant gene NPTII. The samples marked in the red box were the positive T1 GhCIPK6a transgenic plants. M, D2000 DNA ladder; 1–11, positive individuals: 11 J100–1, 11 J100–2, 11 J100–7, 11 J100–8, 11 J100–12, 11 J100–15, 11 J100–21, 11 J100–24, 11 J100–27, 11 J100–34, 11 J100–35, respectively. CK+: positive control. The samples out of the red box were not involved here. B. Southern blotting assay of transgenic cotton plants using the resistance gene NPTII as the probe. In the red box: M, D2000 DNA ladder; 1, OE1 (12D44); 2, OE2 (12D47), which were the sample used in present study. 3–8, represents different individuals, which were 12D48-12D53, respectively. C and D. The genealogical diagram of OE1 (12D44) and OE2 (12D47) lines and their offspring. [file 12870_2020_2548_MOESM8_ESM.docx]

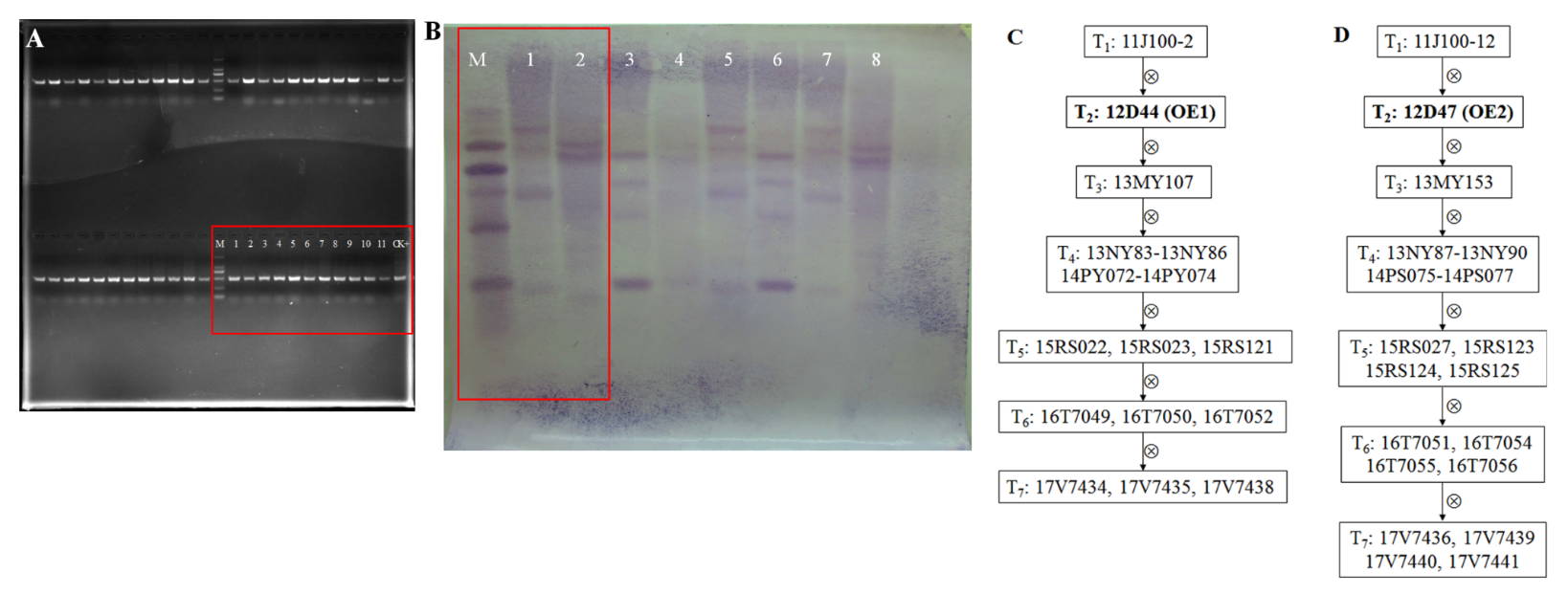


**Additional file 8 Figure S3.** PCR analysis and Southern blotting assay of positive transgenic plants. **A**. Identification of positive T_1_ transgenic plants by amplifying the resistant gene *NPT*II. The samples marked in the red box were the positive T_1_ *GhCIPK6a* transgenic plants. M, D2000 DNA ladder; 1-11, positive individuals: 11J100-1, 11J100-2, 11J100-7, 11J100-8, 11J100-12, 11J100-15, 11J100-21, 11J100-24, 11J100-27, 11J100-34, 11J100-35, respectively. CK+: positive control. The samples out of the red box were not involved here. **B**. Southern blotting assay of transgenic cotton plants using the resistance gene *NPT*II as the probe. In the red box: M, D2000 DNA ladder; 1, OE1 (12D44); 2, OE2 (12D47), which were the sample used in present study. 3-8, represents different individuals, which were 12D48-12D53, respectively. **C** and **D**. The genealogical diagram of OE1 (12D44) and OE2 (12D47) lines and their offspring.
