# Supplementary material for: GhCIPK6a increases salt tolerance in transgenic upland cotton by involving in ROS scavenging and MAPK signaling pathways
Source: BMC Plant Biol. 2020 Sep 14;20:421. doi: 10.1186/s12870-020-02548-4 (PMC7488661; doi:10.1186/s12870-020-02548-4)
Supplement: Supplementary file 9 — Additional file 9: Figure S4. Expression profiles of GhCIPK6s under abiotic stress using public datasets. I, public data from PLEXdb. II, GSE50770, analyzed the crosstalk between genes that are responsive to multiple abiotic stresses, including ABA, cold, drought, salinity, and alkalinity (pH) in G. hirsutum. III, Transcriptome analysis between roots of the salt-tolerant cultivar ‘Zhong 07’ and salt-sensitive cultivar ‘Zhong G5’ after 3, 12, and 48 h of 150 mmol·L− 1 NaCl treatment. [file 12870_2020_2548_MOESM9_ESM.docx]

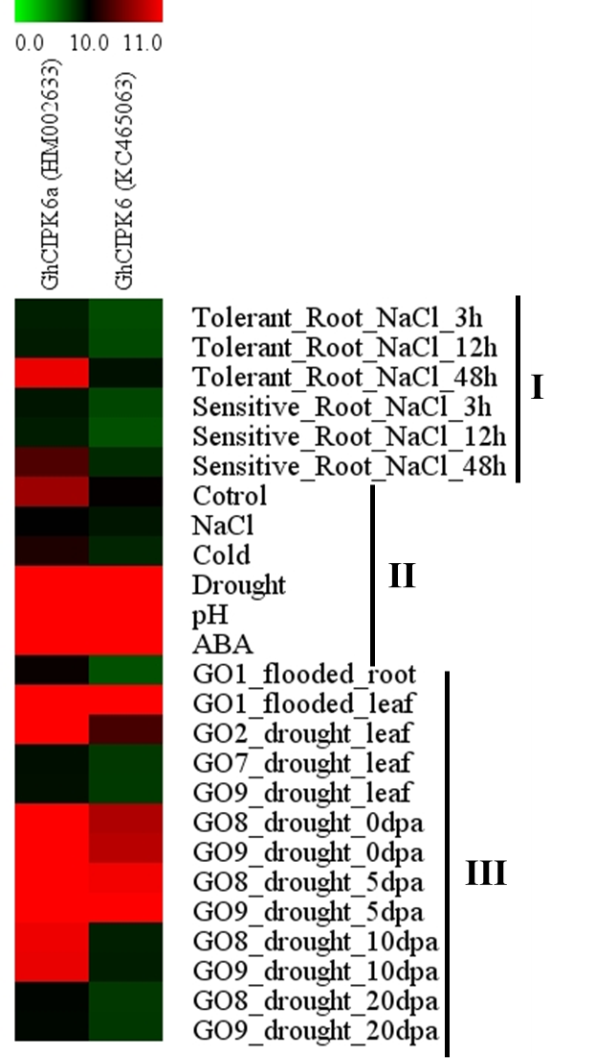


**Additional file 9 Figure S4.** Expression profiles of *GhCIPK6s* under abiotic stress using public datasets. I, public data from PLEXdb. II, GSE50770, analyzed the crosstalk between genes that are responsive to multiple abiotic stresses, including ABA, cold, drought, salinity, and alkalinity (pH) in *G. hirsutum*. III, Transcriptome analysis between roots of the salt-tolerant cultivar ‘Zhong 07’ and salt-sensitive cultivar ‘Zhong G5’ after 3, 12, and 48 h of 150 mmol·L^-1^ NaCl treatment.
