# Supplementary material for: GhCIPK6a increases salt tolerance in transgenic upland cotton by involving in ROS scavenging and MAPK signaling pathways
Source: BMC Plant Biol. 2020 Sep 14;20:421. doi: 10.1186/s12870-020-02548-4 (PMC7488661; doi:10.1186/s12870-020-02548-4)
Supplement: Supplementary file 10 — Additional file 10: Figure S5. The germination performance of transgenic lines (OE1 and OE2) and wild type (WT) line under salt treatment (NaCl) and control (CK) conditions. Bar = 1 cm. [file 12870_2020_2548_MOESM10_ESM.docx]

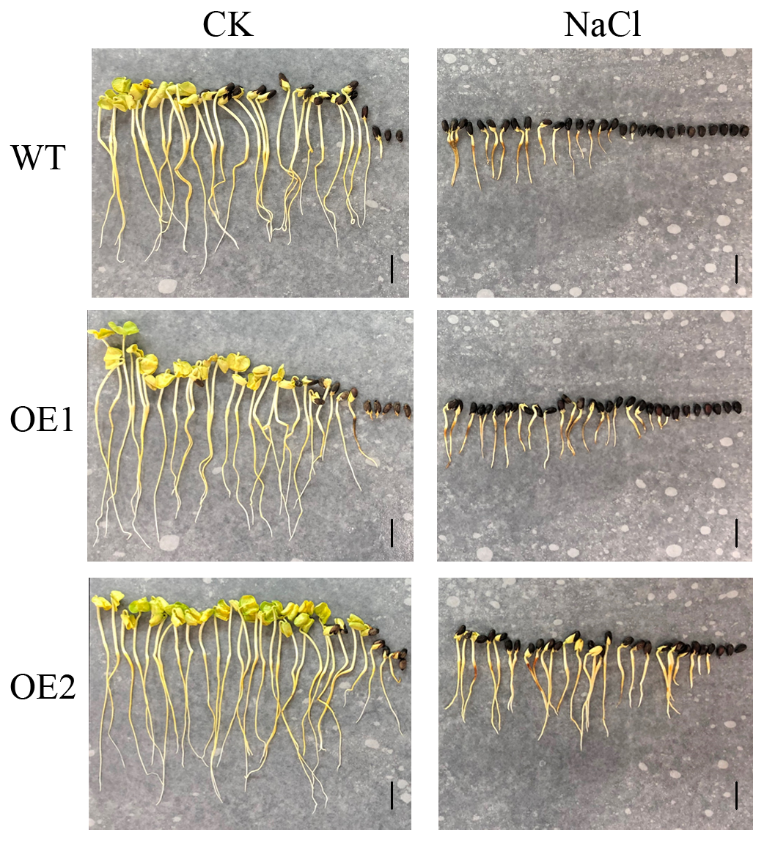


**Additional file 10 Figure S5.** The germination performance of transgenic lines (OE1 and OE2) and wild type line (WT) under salt treatment (NaCl) and control (CK) conditions. Bar = 1 cm.
