# Supplementary material for: GhCIPK6a increases salt tolerance in transgenic upland cotton by involving in ROS scavenging and MAPK signaling pathways
Source: BMC Plant Biol. 2020 Sep 14;20:421. doi: 10.1186/s12870-020-02548-4 (PMC7488661; doi:10.1186/s12870-020-02548-4)
Supplement: Supplementary file 11 — Additional file 11: Figure S6. Salt tolerance assay of transgenic and control cottons during flowering and boll setting stage in the field in Akesu, Xinjiang Autonomous Region, China in 2013. The salt content of the soil under the surface 5 to 10 cm was approximately 0.92%. [file 12870_2020_2548_MOESM11_ESM.docx]

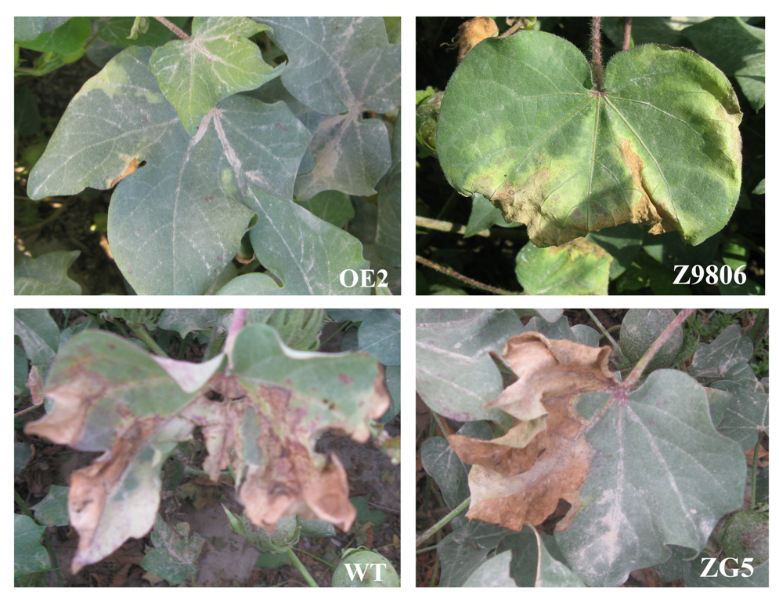


**Additional file 11 Figure S6.** The leaves damaged under salt stress of transgenic and control cotton lines during flowering and boll setting stage in the field in Akesu, Xinjiang Autonomous Region, China in 2013. The salt content of the soil under the surface 5 to 10 cm was approximately 0.92%. WT, wild type line, 11-0516; Z9806, a salt resistant cotton variety; ZG5, a salt sensitive cotton variety.
