# Supplementary material for: GhCIPK6a increases salt tolerance in transgenic upland cotton by involving in ROS scavenging and MAPK signaling pathways
Source: BMC Plant Biol. 2020 Sep 14;20:421. doi: 10.1186/s12870-020-02548-4 (PMC7488661; doi:10.1186/s12870-020-02548-4)
Supplement: Supplementary file 12 — Additional file 12: Figure S7. Venn diagrams of up- and down-regulated DEGs among different salt stress time points in between OE2 and WT plants, identified from RNA-seq analysis. [file 12870_2020_2548_MOESM12_ESM.docx]

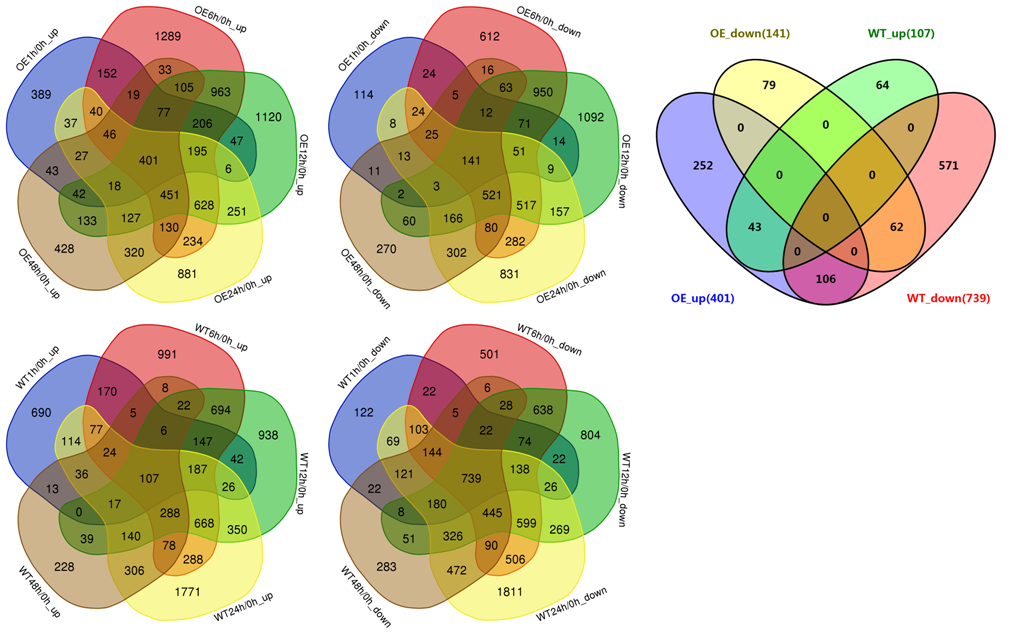


**Additional file 12 Figure S7.** Venn diagrams of up- and down-regulated DEGs among different salt stress time points in between OE and WT plants, identified from RNA-seq analysis.
