# Supplementary material for: GhCIPK6a increases salt tolerance in transgenic upland cotton by involving in ROS scavenging and MAPK signaling pathways
Source: BMC Plant Biol. 2020 Sep 14;20:421. doi: 10.1186/s12870-020-02548-4 (PMC7488661; doi:10.1186/s12870-020-02548-4)
Supplement: Supplementary file 13 — Additional file 13: Figure S8. GO-term and KEGG analysis of up- and down-regulated DEGs. A. GO-term analysis of specific up- and down-regulated DEGs in OE plants following salt treatment. B. KEGG pathway analysis of 78 candidate DEGs. [file 12870_2020_2548_MOESM13_ESM.docx]

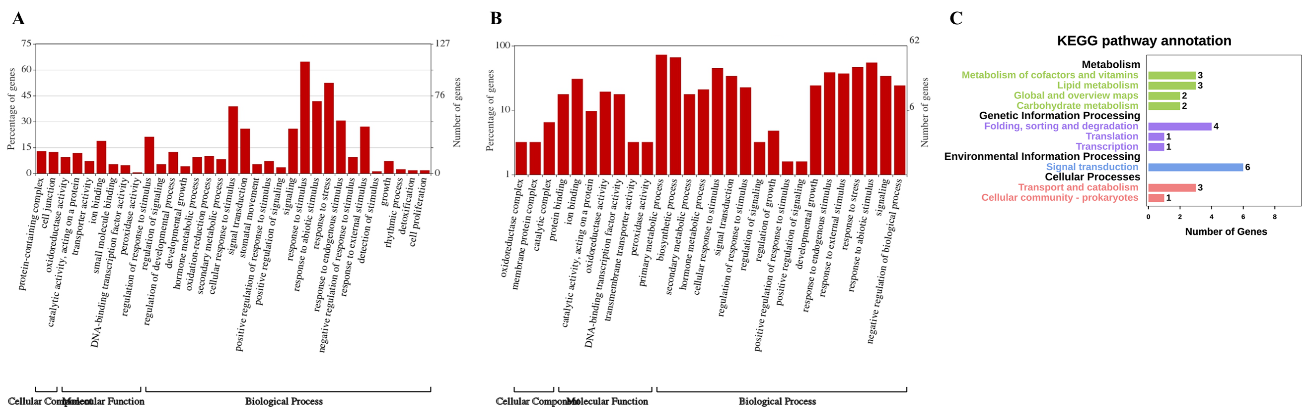


**Additional file 13 Figure S8.** GO-term and KEGG analysis of up- and down-regulated DEGs.

**A.** GO-term analysis of specific up- and down-regulated DEGs in OE plants following salt treatment. **B.** KEGG pathway analysis of 78 candidate DEGs.
