# Supplementary material for: GhCIPK6a increases salt tolerance in transgenic upland cotton by involving in ROS scavenging and MAPK signaling pathways
Source: BMC Plant Biol. 2020 Sep 14;20:421. doi: 10.1186/s12870-020-02548-4 (PMC7488661; doi:10.1186/s12870-020-02548-4)
Supplement: Supplementary file 14 — Additional file 14: Figure S9. Validation of express profiles of candidate DEGs by qRT-PCR. A. RNA-seq analysis and qRT-PCR analysis of the co-expressed DEGs in PPI network. B. Correlation of the expression profiles between RNA-seq and qRT-PCR analysis. [file 12870_2020_2548_MOESM14_ESM.docx]

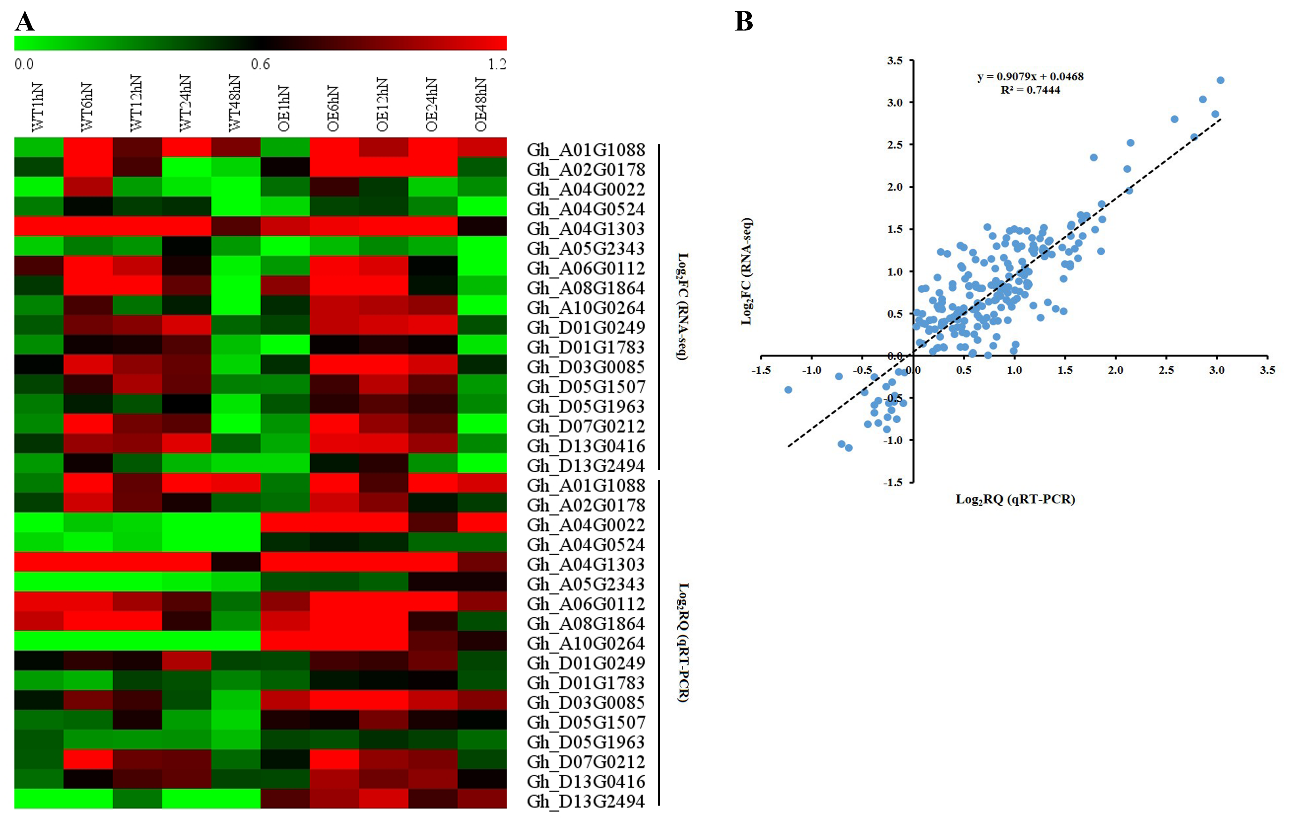


**Additional file 14 Figure S9.** Validation of express profiles of candidate DEGs by qRT-PCR.

**A.** RNA-seq analysis and qRT-PCR analysis of the co-expressed DEGs in PPI network. **B.** Correlation of the expression profiles between RNA-seq and qRT-PCR analysis.
