# Supplementary material for: GhCIPK6a increases salt tolerance in transgenic upland cotton by involving in ROS scavenging and MAPK signaling pathways
Source: BMC Plant Biol. 2020 Sep 14;20:421. doi: 10.1186/s12870-020-02548-4 (PMC7488661; doi:10.1186/s12870-020-02548-4)
Supplement: Supplementary file 15 — Additional file 15: Figure S10. Analysis of differences in amino acid sequences and phosphorylation sites between GhCIPK6a (HM002633) and GhCIPK6 (KC465063). A. Sequence alignment of GhCIPK6a (HM002633) and GhCIPK6 (KC465063). B. Schematic of predicted phosphorylation sites of GhCIPK6s by KinasePhos (http://kinasephos.mbc.nctu.edu.tw/). [file 12870_2020_2548_MOESM15_ESM.docx]

**
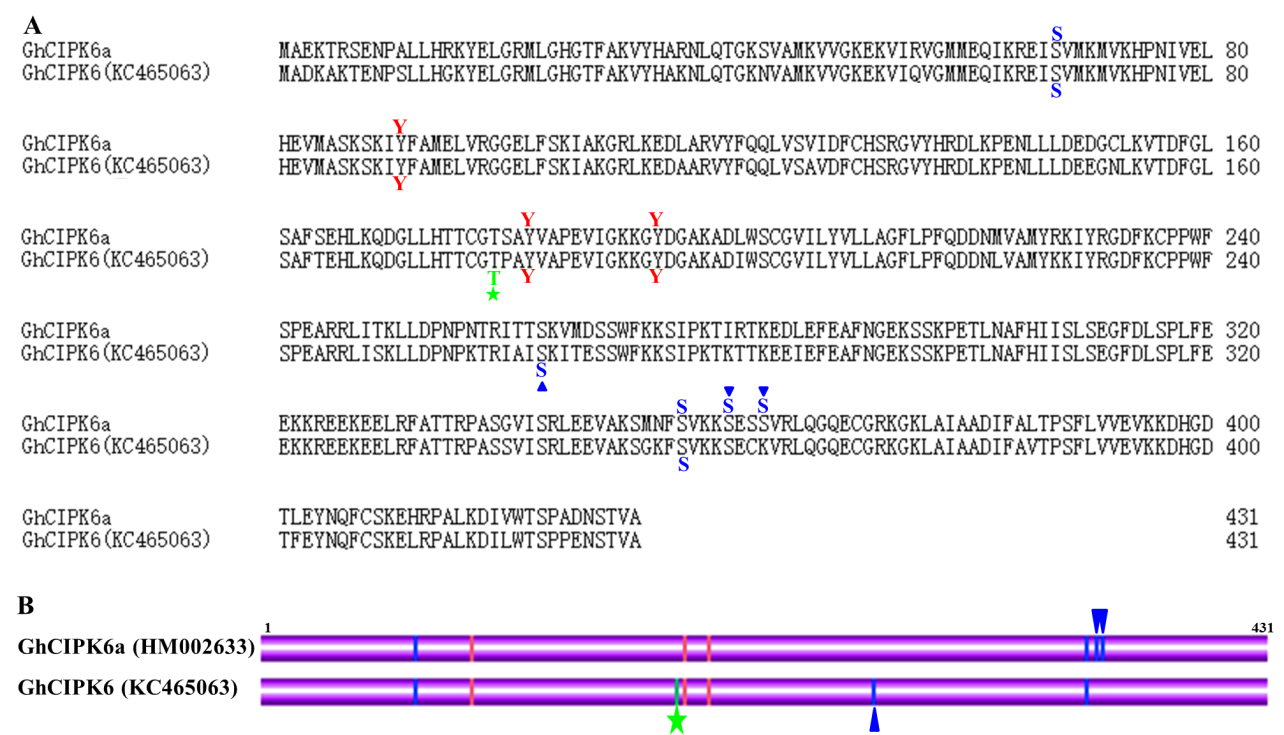
**

**Additional file 15 Figure S10.** Analysis of differences in amino acid sequences and phosphorylation sites between GhCIPK6a (HM002633) and GhCIPK6 (KC465063). **A.** Sequence alignment of GhCIPK6a (HM002633) and GhCIPK6 (KC465063). **B.** Schematic of predicted phosphorylation sites of GhCIPK6s by KinasePhos (http://kinasephos.mbc.nctu.edu.tw/). The predicted phosphorylation sites were labelled with letters. Serine was represented by “S”, threonine by “T”, and tyrosine by “Y”. Differences in predicted phosphorylation sites between GhCIPK6a (HM002633) and GhCIPK6 (KC465063) are marked with geometric shapes. Differences in serine residues are marked with a blue triangle, and differences in threonine residues are marked with a green pentacle.
